# The role of semen and seminal plasma in inducing large-scale genomic changes in the female porcine peri-ovulatory tract

**DOI:** 10.1038/s41598-020-60810-z

**Published:** 2020-03-19

**Authors:** M. Álvarez-Rodríguez, C. A. Martinez, D. Wright, H. Rodríguez-Martinez

**Affiliations:** 1 Department of Biomedical and Clinical Sciences, Faculty of Medicine and Health Sciences, Linköping University, SE-58185 Linköping, Sweden; 2 Department of Physics, Chemistry and Biology, Faculty of Science and Engineering; Linköping University, SE-58183 Linköping, Sweden

**Keywords:** Microarray analysis, Animal breeding

## Abstract

Semen modifies the expression of genes related to immune function along the porcine female internal genital tract. Whether other pathways are induced by the deposition of spermatozoa and/or seminal plasma (SP), is yet undocumented. Here, to determine their relative impact on the uterine and tubal transcriptomes, microarray analyses were performed on the endocervix, endometrium and endosalpinx collected from pre-ovulatory sows 24 h after either mating or artificial insemination (AI) with specific ejaculate fractions containing spermatozoa or sperm-free SP. After enrichment analysis, we found an overrepresentation of genes and pathways associated with sperm transport and binding, oxidative stress and cell-to-cell recognition, such as PI3K-Akt, FoxO signaling, glycosaminoglycan biosynthesis and cAMP-related transcripts, among others. Although semen (either after mating or AI) seemed to have the highest impact along the entire genital tract, our results demonstrate that the SP itself also modifies the transcriptome. The detected modifications of the molecular profiles of the pre/peri-ovulatory endometrium and endosalpinx suggest an interplay for the survival, transport and binding of spermatozoa through, for instance the up-regulation of the Estrogen signaling pathway associated with attachment and release from the oviductal reservoir.

## Introduction

A successful establishment and maintenance of pregnancy is highly dependent on uterine receptivity^1^. Such receptivity implies a reciprocal communication between gametes and the uterine or oviductal epithelia is a key factor for fertilization, embryo development and pregnancy success^2,3^. Understanding the modifications of female genital molecular profiles triggered by seminal components that interact with the internal genital lining during the peri-ovulatory period could be of great importance to overcome problems associated with fertility. Several studies have described the transcriptome changes in the pig uterus and oviduct motivated by the presence of gametes in the female genital tract^4^.

Semen deposited into the female genital tract elicits an immediate inflammatory response^5^. It also activates modifications of genes involved in immune mechanisms^6^ that will promote a state of immune tolerance to paternal antigens in the female^7^. Sperm presence in the oviduct elicits different transcriptomic responses by modulating specific signaling pathways depending on which sex chomosome the spermatozoa contain^4^. Moreover, spermatozoa are suggested to modulate the uterine milieu against oxidative stress to protect spermatozoa and embryos from ROS-induced damage^8^. Presence of spermatozoa and the following sperm-oocyte interactions influence gene expression in the ampullar-isthmic section of the oviduct, changes that would regulate several processes associated with fertilization and embryo development^9^.

However, despite spermatozoa modifying the uterine and/or oviductal environment, there is emerging evidence that the SP interaction with the female genital tract also plays a role in the different events that take place surrounding ovulation and fertilization. The SP has the potential to accelerate ovulation by modifying the endocrine-immune-cytokine network in pre-ovulatory follicles; leading to a reduction in the interval between the LH peak and ovulation time-point^10^. The SP has also been shown to promote subtle changes in the uterine gene expression associated with sperm transport and protection^11^. Furthermore, the SP seems to elicit changes in the expression of certain genes in the oviductal sperm reservoir: the utero-tubal junction^12^.

The study presented here aimed to determine which genes and signaling pathways are expression-modified along the peri-ovulatory female pig genital tract (endocervix, endometrium and endosalpinx) 24 h after the entry of semen and/or sperm-free SP. Since the pig ejaculate is expelled in easily identifiable fractions^5^, where the 1^st^ 10 mL of the sperm-rich fraction (SRF) hereby named P1-fraction contains around 25% of the total spermatozoa of the entire ejaculate mainly immersed in fluid from the epididymis cauda^5^, comparisons were made for the entire ejaculate or this P1-fraction either containing spermatozoa and seminal plasma (Mating: entire ejaculate vs P1-AI: cauda epididymides sperm-peak fraction) or their sperm-free counterparts (SP-Ejac vs SP-P1).

## Results

### Differential gene expression is induced by semen (M or P1-AI) and sperm-free SP (SP-Ejac or SP-P1) through the entire female genital tract

**Figure 1:**
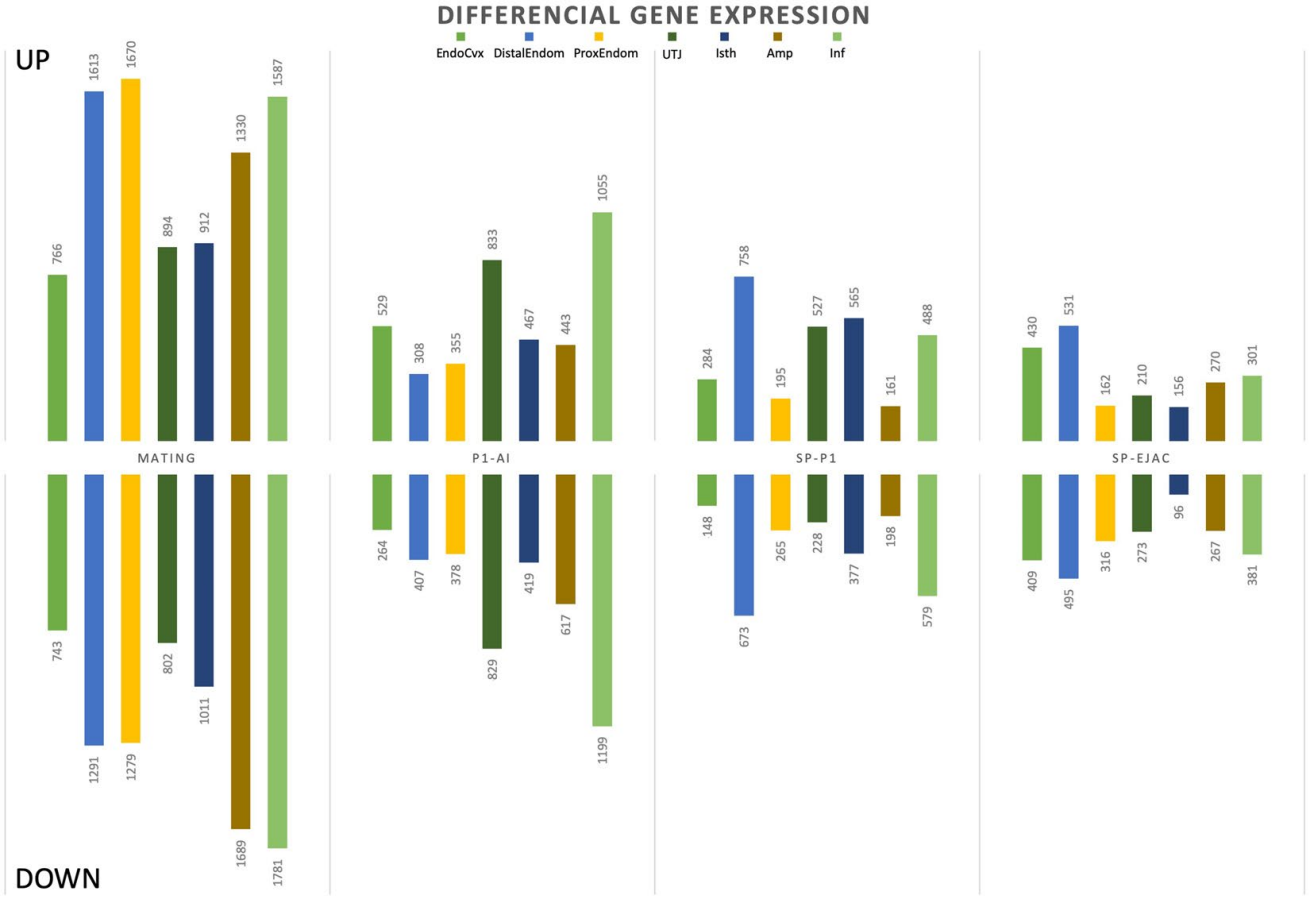
Differential expression (up- and down-regulation) of annotated genes in the mucosa of the internal genital tract of sows after Mating or artificial deposition of the sperm-peak portion P1 (P1-AI); sperm-free seminal plasma of the whole ejaculate (SP-Ejac) or of pooled P1 (SP-P1). All treatments were compared to Control (AI with 50 mL of BTS). The numbers represent the number of differentially expressed genes (p-value < 0.05). Endocervix (EndoCvx), endometrium (distal: DistEndom or proximal: ProxEndom) and endosalpinx from 4 different segments; the utero-tubal junction (UTJ), isthmus (Isth), ampulla (Amp) and infundibulum (Inf).

Following statistical analysis of the data provided by the Affymetrix microarray, we found that an exposure to semen modified the expression of a range of transcripts (1,509–3,369 (M) and 715–2,255 (P1-AI) transcripts) while sperm-free SP treatment altered fewer genes (SP-P1: 359–1,431; SP-Ejac: 252–1026) depending on the section of the genital tract assessed (Table 1). Figure 1 shows the numbers of genes differentially expressed in the mucosal samples collected along the sow internal genital tract. Overall, the highest differences were found in the M-group, compared to the rest of the experimental groups among all mucosal segments, with the exception of the utero-tubal junction (UTJ) where the total number of differentially expressed genes was similar between M- and P1-AI semen groups (1,696 vs. 1,662 genes, respectively).

**Table 1:** Differential expression (up- and down-regulation) among treatments of total genes included in the microarrays (25,470) in the different segments of the internal genital tract of sows after mating (M) or artificial deposition of the sperm-peak portion P1 (P1-AI); sperm-free seminal plasma of from P1-fraction P1 (SP-P1) or of the whole ejaculate (SP-Ejac).

	M	M (%)	P1-AI	P1-AI (%)	SP-P1	SP-P1 (%)	SP-Ejac	SP-Ejac (%)
**EndoCvx**	1509	5.9	793	3.1	432	1.7	839	3.3
**DistEndom**	2905	11.4	715	2.8	1431	5.6	1026	4
**ProxEndom**	2949	11.6	733	2.9	460	1.8	478	1.9
**UTJ**	1696	6.7	1662	6.5	755	3	483	1.9
**Isth**	1923	7.6	886	3.5	942	3.7	252	1
**Amp**	3028	11.9	1060	4.2	359	1.4	537	2.1
**Inf**	3369	13.2	2255	8.9	1068	4.2	682	2.7

All treatments were compared to Control (AI with 50 mL of BTS).

### Commonly altered genes among mucosal segments within each experimental group

**Figure 2:**
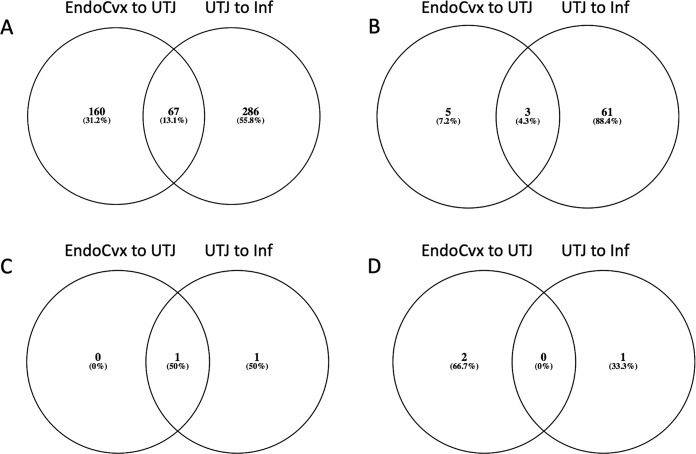
Venn diagrams depicting common genes among mucosal segments of the internal genital tract of the sow, after the various treatments: M (**A**), P1-AI (**B**), SP-P1 (**C**) and SP-Ejac (**D**).

Each treatment induced transcriptomic changes that included a particular subset of genes common to all mucosal segments (the number of these common genes is shown in the Venn diagrams in Fig. 2). The M-treatment induced the largest number of altered genes common to all mucosal segments **(**19 genes UP- & 47 genes DOWN), followed by P1-AI (2 genes UP- & 1 genes DOWN), and SP-P1 (1 gene UP- & 0 genes DOWN). We did not find altered genes common within all mucosal segments of the SP-Ejac group. The names of the genes represented in the Venn diagrams are shown in Table 2.

**Table 2:** Identification of the common genes among all tissues represented in Venn diagrams from Fig. 2.

M		P1-AI		SP-P1		SP-Ejac	
UP (19)	DOWN (48)	UP (2)	DOWN (1)	UP (1)	DOWN (0)	UP (0)	DOWN (0)
*ABCA9*, *FAM107A*, *FIGF*, *FMO1*, *FMO2*, *GAB1*, *CCDC68*, *MICAL3,NDRG4*, *SLC2A12*, *LOC106504056*, *EZH1*, *PPIP5K1*, *DECR2*, *ALDH6A1*, *CHDH*, *LOC100522894*, *GLUL*, *NCOA2*	*LOC100523107*, *FAM98A*, *CALR*, *VIMP*, *MIR671*, *EMC3*, *CALU*, *CPQ*, *SNX31*, *TMEM263*, *SEC. 23A*, *TPM4*, *RAI14*, *ARF4*, *TCEAL8*, *OSTC*, *B4GALT1*, *LOC100514264*, *RCN1*, *DNAJB11*, *ABHD2*, *GPX8*, *TSPAN6*, *HSPA13*, *FKBP4*, *KDELR3*, *TRPC5*, *LPPR5*, *FRD1*, *FAM69A*, *LOC100624484*, *SNAI2*, *REXO2*, *TDG*, *OLFML3*, *FAM19A4*, *HTR2A*, *CLRN3*, *DLGAP2*, *SRPX2*, *OLFM3*, *ENPEP*, *TAC3*, *PLAUR*, *VCAN*, *FGFBP1*, *HTRA4*	*AOAH* *LOC102162715*	*KIT*	*NMNAT1*			

Mating (M) or artificial deposition of the sperm-peak portion P1 (P1-AI); sperm-free seminal plasma of from P1-fraction P1 (SP-P1) or of the whole ejaculate (SP-Ejac). All treatments were compared to Control (AI with 50 mL of BTS).

### Biological meaning of the differentially expressed transcripts

#### Analysis of functional categories

**Figure 3:**
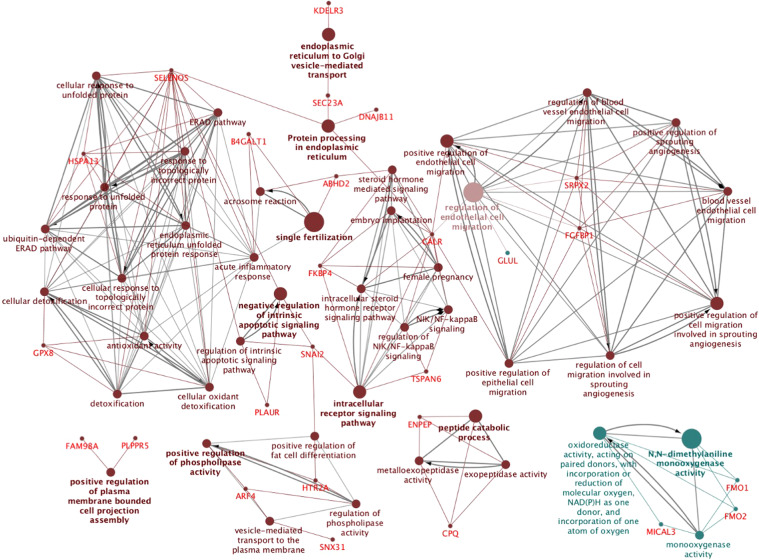
Schematic representation of common altered transcripts among all mucosal tissues collected from the M group. The analysis of overrepresented functional categories was performed using the Cytoscape v3.0.0 application ClueGo v2.0.3. Terms are functionally grouped based on shared genes (kappa score) and are shown in different colors. The size of the nodes indicates the degree of significance, where the biggest nodes correspond to highest significance. The following ClueGo parameters were used: GO tree levels, 3–6 (first level = 0); minimum number of genes, 1; P-value correction, Benjamini-Hochberg, terms with P < 0.05, GO term connection restriction (kappa score), 0.4; GO term grouping, initial group size of 1. The resulting network was modified; that is, some redundant and non-informative terms were deleted and the network manually rearranged.

**Figure 4:**
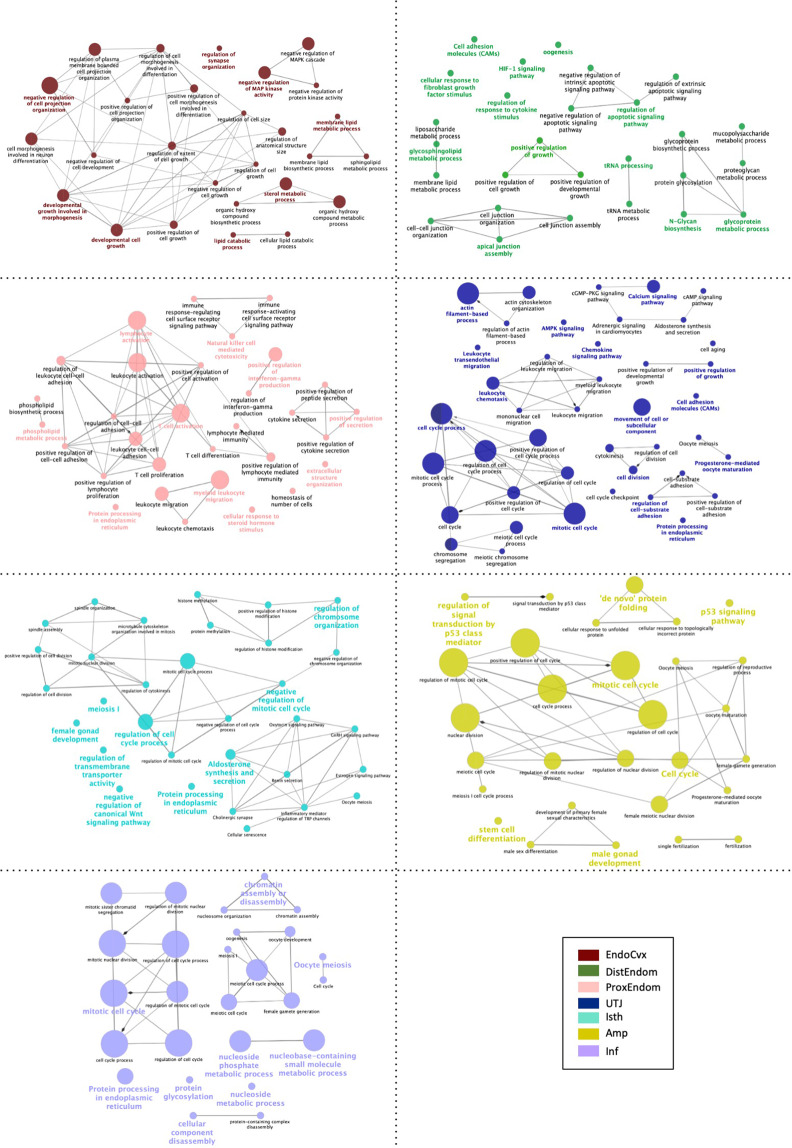
Schematic representation of selected altered transcripts in the mucosae of segments of the internal genital tract of the sows in P1-AI group; Endocervix (EndoCvx: (**A**)), Endometrium (DisEndom: (**B**), ProEndom: (**C**)), Utero-tubal Junction (UTJ: (**D**)), Isthmus (Isth: (**E**)), Ampulla (Amp: (**F**)) and Infundibulum (Inf: (**G**)). The analysis of overrepresented functional categories was performed using the Cytoscape v3.0.0 application ClueGo v2.0.3. Terms are functionally grouped based on shared genes (kappa score) and are shown in different colors. The size of the nodes indicates the degree of significance, where the biggest nodes correspond to highest significance. The following ClueGo parameters were used: GO tree levels 3–6 (first level = 0); minimum number of genes 1; minimum percentage of genes 8, 5P-value correction, Benjamini-Hochberg, terms with P < 0.05, GO term connection restriction (kappa score) 0.4; GO term grouping, GO term fusion; initial group size of 3. The resulting network was modified; that is, some redundant and non-informative terms were deleted and the network manually rearranged.

**Figure 5:**
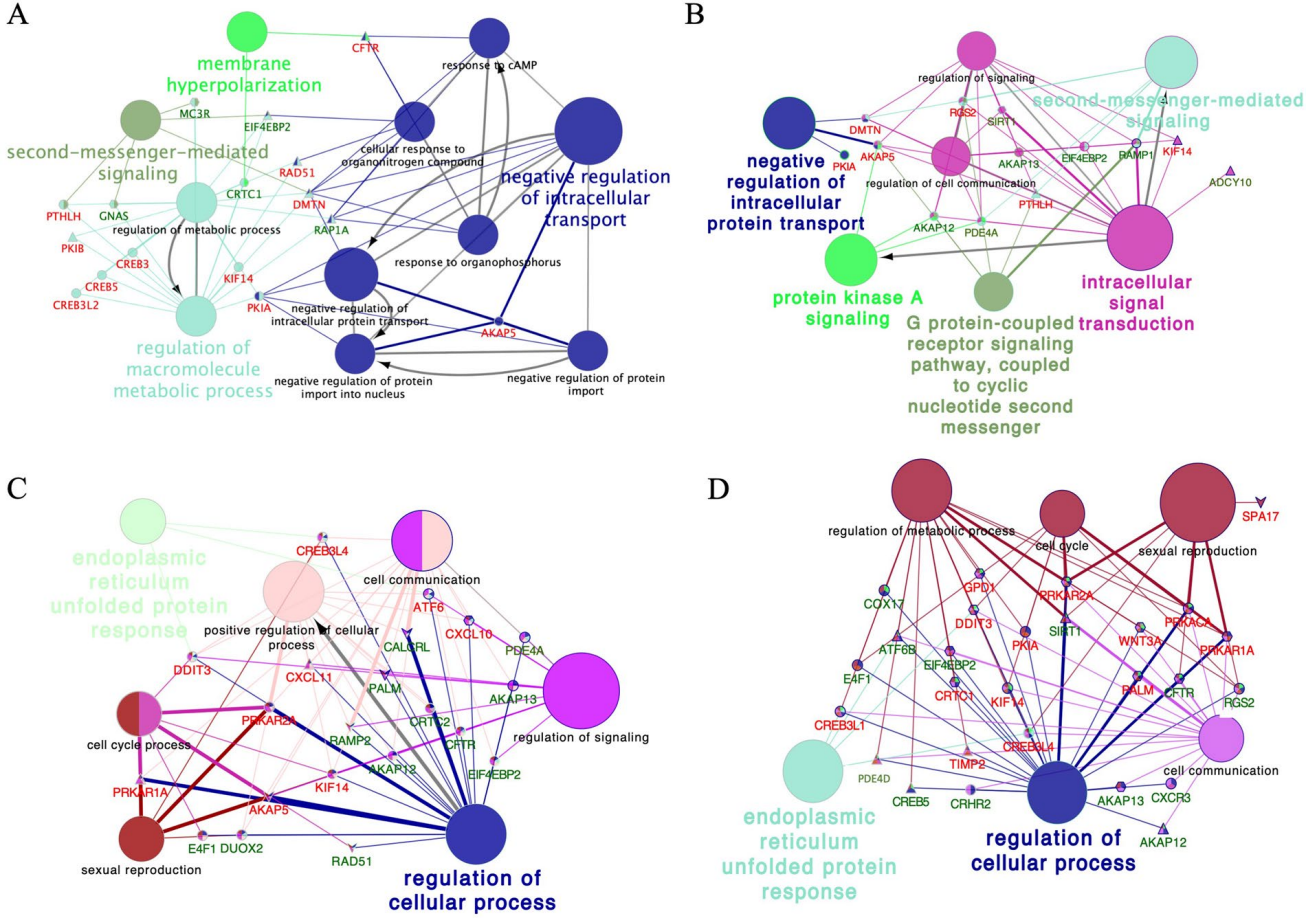
Schematic representation of CAMP-related transcripts in the mucosa of the segments of the sow oviduct [Utero-tubal junction (UTJ: (**A**)), Isthmus (Isth: (**B**)), Ampulla (Amp: (**C**)) and Infundibulum (Inf: (**D**))] after mating (M: Elipse); Artificial insemination of the sperm-peak fraction (P1-AI: Triangle), seminal plasma from the sperm-peak fraction (SP-P1: Hexagon); or seminal plasma from the entire ejaculate (SP-Ejac: V). The analysis of overrepresented functional categories was performed using the Cytoscape v3.0.0 application ClueGo v2.0.3. Terms are functionally grouped based on shared genes (kappa score) and are shown in different colors. The size of the nodes indicates the degree of significance, where the biggest nodes correspond to highest significance. The following ClueGo parameters were used: GO tree levels, 1–4 (first level = 0); minimum number of genes, 1; P-value correction, Benjamini-Hochberg, terms with P < 0.05, GO term connection restriction (kappa score), 0.4; GO term grouping, initial group size of 1. The resulting network was modified; that is, some redundant and non-informative terms were deleted and the network manually rearranged.

Since the M-group induced the largest number of transcriptomic changes among all tissues relative to the rest of the experimental groups, we performed an overall representation of the most significantly altered biological terms along the entire internal genital tract of the sow, where we found evidence of overrepresentation of processes involved in reproduction such as: single fertilization, acrosome reaction, steroid biosynthesis, and female pregnancy, amongst others (Fig. 3). Figure 4 shows the functional categories of altered genes in the P1-AI group from all mucosal segments analyzed (Endocervix (EndoCvx) to Infundibulum (Inf)) where many cellular processes were consistently represented. Interestingly, the oviduct segments (Isthmus utero-tubal junction (Isth) to Infundibulum (Inf)) identified a high number of GO terms associated with gamete function, oocyte meiosis, male and female gonadal development, male sex differentiation, etc. The most relevant GO terms found in the sperm-free SP groups are shown in Table 3, where the largest number of alterations are found in the SP-Ejac group, while the SP-P1 group identified functions related with sperm survival and apoptosis. Additional gene ontology (GO) analysis of altered cAMP-related transcripts in all experimental groups along the oviduct is presented in Fig. 5A–D (A, UTJ); (B, Isth); (C, Ampulla (Amp)); (D, Inf), depicting the consistent overrepresentation of cAMP-related genes throughout, highly relevant to sperm motility, the main functional mechanism for sperm release from the oviduct reservoir pending fertilization.

**Table 3:** Biological function of differentially expressed genes (Up & Down regulated) in sperm-free groups among the entire female genital tract of the sow.

Genes	Tissue	Group	Expression	Biological role
*GAPDH*	**Inf**	SP-P1	DOWN	Inition of apoptosis
*ATM*	**DistEndom**	SP-P1; SP-Ejac	UP	Phosphorylation of key proteins involved in DNA repair and apoptosis
	**Isth**	SP-P1	UP	
*IFN-alpha9*	**ProxEndom**	SP-P1	DOWN	Activation of immune system
	**ProxEndom**	SP-Ejac	UP	
*PRLR*	**ProxEndom**	SP-P1; SP-Ejac	DOWN	Prosurvival factor for spermatozoa by inhibiting sperm capacitation through suppression of SRC kinase activation and stimulation of AKT
*BCL6*	**DistEndom**	SP-Ejac	DOWN	Modulation of IL-4 and lead the differentiation of naive helper T-cells
*CDKN2D*	**DistEndom**	SP-Ejac	DOWN	Prevents the activation of CDK kinases
*G6PC2*	**DistEndom**	SP-Ejac	DOWN	Catalyzes the final steps of gluconeogenic and glycogenolytic pathways
*KRAS*	**DistEndom**	SP-Ejac	UP	Controls cell proliferation
*PRKAB1*	**DistEndom**	SP-Ejac	UP	Activated protein kinase subunit beta-1
*ANAPC13*	**DistEndom**	SP-Ejac	UP	Controls the G1 phase of the cell cycle
*HDAC2*	**DistEndom**	SP-Ejac	UP	Involved in cell cycle progression
*COL5A3*	**ProxEndom**	SP-Ejac	DOWN	Collagen alpha-3(V) chain, of the low abundance fibrillar collagens
*CSF3*	**ProxEndom**	SP-Ejac	DOWN	Act in hematopoiesis by controlling the production, differentiation, and function of 2 related white cell populations of the blood, the granulocytes and the monocytes-macrophages
*TSC2*	**ProxEndom**	SP-Ejac	DOWN	Stimulate GTPases. Interacts with Hsp70 and Hsp90
*CHST7*	**EndoCvx**, **Dist endom**	SP-Ejac	DOWN	Important determinant of sulfated GAGs expression and the associated function of chondroitin sulfates as regulators of many biologic processes
*CHST3*	**Endo cvx**	SP-Ejac	DOWN	Related with GAGs as well as CHST7
*CHST12*	**DistEndom**	SP-Ejac	DOWN	Related with GAGs as well as CHST7
*DSE*	**EndoCvx**	SP-Ejac	DOWN	Induce T lymphocytes
*E2F3*	**DistEndom**	SP-Ejac	DOWN	Transcription factor, control cycle
*STAG2*	**DistEndom**	SP-Ejac	UP	Part of the cohesion comples to hold the chromatid togheter
*FGFR1*	**ProxEndom**	SP-Ejac	DOWN	Indirect activation of the gamma isoforms of phospholipase C (PLCγ)
*PPP2R5D*	**ProxEndom**	SP-Ejac	UP	Negative control of cell growth and division

Sperm-free seminal plasma of from P1-fraction P1 (SP-P1) or of the whole ejaculate (SP-Ejac). All treatments were compared to Control (AI with 50 mL of BTS).

#### Pathway analysis

We selected among differently expressed biological pathways depending on their statistical significance (P < 0.05) in regards to the different treatments and mucosal segments of the internal genital tract (Tables 4–7**)**. We found many altered pathways with important roles in reproductive-related processes such as the phosphatidylinositol 3-kinase (PI3K-Akt) pathway, which was altered in the M-group (distal and proximal endometria (DistEndom, ProxEndom) and Amp) and also in the SP-Ejac (ProxEndom) and SP-P1 (ProxEndom and Amp) groups (Tables 4, 6 and 7), the FoxO signaling pathway, modified by M (Amp and Inf), P1-AI (Inf), SP-Ejac (DistEndom) and SP-P1 (Inf) (Tables 3–6**)**, or the N-glycans biosynthesis, that was enriched after M (UTJ, Amp and Inf), P1-AI (EndoCvx, Isth and Inf), and SP-P1 (EndoCvx) (Tables 4, 5 and 7). The Estrogen signaling pathway appeared to be mainly altered after the sperm-free SP treatment SP-P (UTJ and Isth) (Table 7). The Supplementary figures 1 to 14 depict a detailed view of the up- and down-regulation of genes according to Biological and Molecular functions. In general, for biological functions, Binding and Catalytic activity were constantly overrepresented in all mucosal segments, independently of the experimental group. In the molecular function analysis, the cellular process of biological regulation, the response to stimulus developmental process and metabolic processes were overrepresented, independ of both the mucosal segment and experimental group considered.

**Table 4:** List of the most significant differently expressed biological pathways (P < 0.05) examined with KEGG database in mating (M), in comparison with the Control group.

	*PATHWAY ID*	*PATHWAY NAME*	*ENRICHMENT SCORE*	*P-VALUE*	*GENES ALTERED%*	*GENE LIST*
**EndoCvx**						
*1*	KEGG_103	Steroid biosynthesis	6.7	0.001	41.7	*CYP51*, ***FAXDC2***, *MSMO1*, *NSDHL*, *SQLE*
*2*	KEGG_8	Glycosaminoglycan biosynthesis	5.6	0.003	33.3	*CHST14*, *CHST3*, *CHSY1*, *CHSY3*, *DSE*
*3*	KEGG_34	Focal adhesion	5.3	0.005	14.7	*ACTB*, *CAV1*, *CAV2*, *CDC42*, *COL1A2*, *COL3A1*, *COL6A3*, *COL6A3*, ***ERBB2***, *HGF*, ***IGF1R***, *ITGA2*, *ITGB6*, ***MAPK1***, *ROCK2*, *THBS1*, ***THBS3***, ***VEGFB***, *ZYX*
*4*	KEGG_49	Adherens junction	5	0.007	19.6	*ACTB*, *CDC42*, ***CSNK2A2***, ***CSNK2B***, ***ERBB2***, ***IGF1R***, ***INSR***, ***MAPK1***, *SNAI2*
**DistEndom**						
*1*	KEGG_34	Focal adhesion	10.6	0.0002	25.2	*CCND2*, *COL11A1*, *COL1A2*, *COL3A1*, *COL4A1*, *COL4A6*, *COL5A2*, *COL6A3*, *COL6A3*, *COL6A3*, ***ERBB2***, *FLT1*, *IGF1*, ***IGF1R***, *ITGA1*, *ITGA2*, *ITGA5*, *ITGA8*, *ITGAV*, *ITGB6*, *LAMB1*, ***MAPK1***, *MAPK8*, ***PDGFC***, ***PDGFD***, *SHC1*, *THBS1*, ***THBS3***, *TLN2*, *VCL*, *VEGFA*, ***VEGFB***, *ZYX*
*2*	KEGG_213	PI3K-Akt signaling pathway	5.4	0.004	17.9	*CCND2*, *COL11A1*, *COL1A2*, *COL3A1*, *COL4A1*, *COL4A6*, *COL5A2*, *COL6A3*, *COL6A3*, *COL6A3*, *CREB3L1*, *CREB3L2*, *CREB5*, ***CSF1R***, ***CSF3***, *FGF17*, *FGF2*, *FGF20*, *FGFR1*, *FLT1*, *G6PC2*, *IGF1*, ***IGF1R***, *IL7*, *ITGA1*, *ITGA2*, *ITGA5*, *ITGA8*, *ITGAV*, *ITGB6*, *LAMB1*, ***MAPK1***, ***MYB***, ***PDGFC***, ***PDGFD***, *PIK3AP1*, ***PPP2R5D***, ***STK11***, *THBS1*, ***THBS3***, *VEGFA*, ***VEGFB***
*3*	KEGG_8	Glycosaminoglycan biosynthesis	5.2	0.005	40	*CHST12*, *CHST7*, *CHSY1*, *CHSY3*, *CSGALNACT2*, *DSE*
*4*	KEGG_46	Estrogen signaling pathway	3.7	0.02	26.9	***CALM1***, *CREB3L1*, *CREB3L2*, *CREB5*, *FKBP4*, *HBEGF*, *HSPA2*, ***MAPK1***, *MMP2*, *MMP9*, ***PLCB4***, ***PRKACA***, *SHC1*
*5*	KEGG_49	Adherens junction	3.2	0.03	21.7	***ERBB2***, *FGFR1*, ***IGF1R***, ***MAPK1***, *PTPN1*, *PVRL1*, *SNAI2*, *SORBS1*, *TCF7L2*, *VCL*
**ProxEndom**						
*1*	KEGG_34	Focal adhesion	8.8	0.0001	20.3	*BRAF*, *CCND2*, *COL1A2*, *COL3A1*, *COL4A1*, *COL5A2*, *COL6A3*, *COL6A3*, ***COMP***, *CTNNB1*, ***FIGF***, *FLT1*, *HGF*, *IGF1*, ***IGF1R***, *ITGA1*, *ITGA2*, *ITGA8*, *KDR*, ***LAMB1***, ***MYLPF***, *PIK3R5*, *SHC1*, *TLN2*, ***TNR***, *VEGFA*
*2*	KEGG_213	PI3K-Akt signaling pathway	7.8	0.0004	16.5	*ANGPT2*, *CCND2*, *COL1A2*, *COL3A1*, *COL4A1*, *COL5A2*, *COL6A3*, ***COMP***, *CREB3L1*, *CREB3L2*, *CREB3L4*, *CREB5*, ***CSF1R***, ***CSF3***, ***EFNA2***, ***FGF17***, ***FGF21***, ***FGF5***, *FGFR1*, ***FIGF***, *FLT1*, ***G6PC2***, ***GH1***, *HGF*, *IGF1*, ***IGF1R***, *IL7*, *ITGA1*, *ITGA2*, *ITGA8*, *KDR*, *LAMB1*, *PIK3AP1*, ***PIK3R5***, ***STK11***, ***TNR***, *VEGFA*
*3*	KEGG_98	Rap1 signaling pathway	6.3	0.001	17.5	*ANGPT2*, *BRAF*, ***CALM1***, ***CSF1R***, *CTNNB1*, ***EFNA2***, ***FGF17***, ***FGF21***, ***FGF5***, *FGFR1*, ***FIGF***, *FLT1*, *GNAS*, ***GRIN2B***, *HGF*, *IGF1*, ***IGF1R***, *KDR*, ***PIK3R5***, ***PLCB4***, *RAPGEF2*, *SIPA1L1* *TLN2*, *VEGFA*
*4*	KEGG_46	Estrogen signaling pathway	5.6	0.003	21.3	***CALM1***, *CREB3L1*, *CREB3L2*, *CREB3L4*, *CREB5*, *FKBP4*, *GNAS*, *HSPA2*, ***KCNJ3***, *MMP9*, ***PIK3R5***, ***PLCB4***, *SHC1*
*5*	KEGG_105	Cell adhesion molecules	5.4	0.004	18.7	***CDH15***, *CLDN11*, ***CLDN2***, ***CLDN5***, *ITGA8*, ***NRXN2***, *NTNG1*, *PVRL1*, *SDC1*, *SDC2*, *SDC4*, *SELL*, ***SELP***, *SIGLEC1*, *SLA-8*, *VCAM1*, *VCAN*
*6*	KEGG_165	Mucin type O-Glycan biosynthesis	4.5	0.01	28.6	*GALNTL5*, *GCNT1*, *GCNT3*, *GCNT4*, *ST3GAL1*, ***ST6GALNAC1***
*7*	KEGG_8	Glycosaminoglycan biosynthesis	4.2	0.01	36.4	*B3GNT2*, *B4GALT1*, ***CHST4***, *ST3GAL1*
**UTJ**						
*1*	KEGG_103	Steroid biosynthesis	8.7	0.0007	21.4	*CYP51*, ***FAXDC2***, ***LIPA***, *MSMO1*, *NSDHL*, *SQLE*
*2*	KEGG_110	N-glycans biosynthesis	4.8	0.008	21.6	***ALG13***, *B4GALT1*, *B4GALT3*, *DDOST*, *DPM3*, *MAN2A1*, *MGAT4B*, *STT3A*
*3*	KEGG_8	Glycosaminoglycan biosynthesis	3.6	0.02	26.7	*B4GALT7*, *CHST12*, *CHSY3*, *DSE*
**Isth**						
*1*	KEGG_59	Cell cycle	6.7	0.001	20.3	*ANAPC13*, ***ATM***, *CCNB2*, *CCND2*, *CDC20*, *CDC25C*, ***EP300***, *ESPL1*, *FZR1*, ***ORC4***, *PTTG1*, ***RB1***, ***STAG2***, *TP53*, *TTK*
*2*	KEGG_34	Focal adhesion	3.5	0.03	13.8	***BRAF***, *CCND2*, *COL4A1*, *COL5A2*, *CTNNB1*, ***ELK1***, *FIGF*, *HGF*, ***IGF1R***, ***LAMA4***, *MYLK2*, ***PAK1***, *PTEN*, *SHC1*, ***SOS2***, ***SRC***, ***VEGFB***
*4*	KEGG_49	Adherens junction	3.2	0.03	17.4	*CTNNB1*, ***EP300***, ***IGF1R***, *SNAI2*, ***SORBS1***, ***SRC***, *SSX2IP*, ***WASL***
	KEGG_98	Rap1 signaling pathway	3.2	0.04	13.1	***ADORA2A***, ***BRAF***, *CTNNB1*, ***FIGF***, *GNAI2*, ***GNAO1***, *HGF*, ***IGF1R***, ***LPAR2***, ***MAGI3***, ***MAP2K6***, ***MAPK14***, ***P2RY1***, ***PLCB4***, ***RAPGEF6***, ***RASGRP2***, ***SRC***, ***VEGFB***
**Amp**						
*1*	KEGG_59	Cell cycle	12.4	0.0004	32.4	***ABL1***, *ANAPC13*, *BUB3*, *CCNA1*, *CCNB2*, *CCNB3*, *CCNE2*, ***CCNH***, *CDC20*, *CDC23*, ***CDC25B***, *CDC25C*, *CDC27*, *CDK1*, *CHEK1*, *E2F5*, ***EP300***, *HDAC2*, *MCM4*, *MDM2*, *PTTG1*, *TTK*, *YWHAH*, *YWHAZ*
*2*	KEGG_66	FoxO signaling pathway	6.4	0.001	23.7	***AKT2***, ***ARAF***, ***BCL2L11***, ***BCL6***, *CCNB2*, *CCNB3*, *CHUK*, ***EP300***, ***FOXO3***, ***IGF1R***, ***INSR***, *KRAS*, ***MAPK1***, *MAPK8*, *MDM2*, *PCK2*, ***PIK3R5***, *PLK4*, ***RAG1***, ***RAG2***, ***STAT3***, *USP7*
*3*	KEGG_195	HIF-1 signaling pathway	5.3	0.004	23.4	***AKT2***, ***EGLN1***, ***EGLN3***, *EIF4E*, ***EP300***, ***ERBB2***, ***HK1***, *HKDC1*, ***IFNGR1***, ***IGF1R***, ***INSR***, ***MAPK1***, ***NFKB1***, *PDHB*, ***PIK3R5***, ***RELA***, ***STAT3***, *TCEB1*
*4*	KEGG_103	Steroid biosynthesis	4.5	0.01	41.7	***CYP24A1***, *HSD17B7*, *MSMO1*, *NSDHL*, *SQLE*
*5*	KEGG_213	PI3K-Akt signaling pathway	3.8	0.02	17	***AKT2***, ***BCL2L1***, ***BCL2L11***, *CCNE2*, *CHUK*, ***COL5A3***, ***CRTC2***, ***DDIT4***, ***EFNA1***, *EIF4E*, *FGF19*, ***FN1***, ***FOXO3***, ***GHR***, *GNB5*, ***GNG11***, *HGF*, ***IGF1R***, ***INSR***, *ITGA11*, ***ITGB4***, *KRAS*, *LPAR1*, ***LPAR2***, ***LPAR6***, ***MAPK1***, *MDM2*, ***NFKB1***, *PCK2*, ***PIK3R5***, *PPP2R2A*, ***RELA***, ***TNC***, ***TNR***, ***TSC2***, ***VEGFB***, *YWHAH*, *YWHAZ*
*6*	KEGG_110	N-glycans biosynthesis	3.4	0.03	24.3	*ALG14*, *ALG5*, *B4GALT1*, *DAD1*, *DDOST*, *DOLPP1*, ***MAN2A2***, *RPN1*, *STT3A*
**Inf**						
*1*	KEGG_59	Cell cycle	7.3	0.0006	28.4	*ANAPC13*, *BUB3*, *CCNB2*, *CCNB3*, *CCNE2*, *CDC20*, *CDC23*, ***CDC25B***, *CDC25C*, *CDC27*, *CDK1*, ***CDK6***, *CDKN2D*, *E2F3*, ***EP300***, *FZR1*, *HDAC2*, *PTTG1*, ***TGFB2***, *TTK*, *YWHAH*
*2*	KEGG_49	Adherens junction	7.1	0.0008	32.6	*CDC42*, *CSNK2B*, ***CTNNA3***, ***EP300***, ***ERBB2***, ***IGF1R***, ***IQGAP1***, *MAP3K7*, ***MAPK1***, ***MET***, *PTPN1*, ***PVRL4***, *SNAI2*, ***SORBS1***, *SSX2IP*, *TCF7L1*
*3*	KEGG_195	HIF-1 signaling pathway	6.7	0.001	27.3	**ANGPT1, CAMK2D, EGLN1, EGLN3**, EIF4E, **EP300, ERBB2**, GAPDH, **HK1, IFNGR1**, IGF1, **IGF1R, LTBR, MAPK1, MKNK2, NFKB1, NOS2**, PDHB, RPS6, **STAT3, TEK**
*4*	KEGG_66	FoxO signaling pathway	5.8	0.002	24.7	***BCL2L11***, ***BCL6***, *CAT*, *CCNB2*, *CCNB3*, *CDKN2D*, *CHUK*, ***EP300***, ***FOXO4***, ***GABARAP***, *IGF1*, ***IGF1R***, *KRAS*, ***MAPK1***, *PLK4*, *PRKAB1*, *PRMT1*, ***SIRT1***, ***SOS2***, ***STAT3***, ***TGFB2***, ***TNFSF10***, *USP7*
*5*	KEGG_110	N-glycans biosynthesis	4.7	0.008	29.7	*ALG14*, *ALG2*, *ALG5*, *DAD1*, *DDOST*, *DOLPP1*, ***MAN1A1***, ***MAN2A2***, *MGAT2*, *RPN1*, *STT3A*
*6*	KEGG_103	Steroid biosynthesis	4.1	0.01	25.5	***CYP24A1***, *LIPA*, *MSMO1*, *NSDHL*, *SQLE*

*Upregulated genes are marked in bold; the unmarked were downregulated.

Endocervix (EndoCvx), Endometrium (DistEndom, ProxEndom), and endosalpinx from Utero-tubal Junction (UTJ), Isthmus (Isth), Ampulla (Amp) and Infundibulum (Inf).

**Table 5:** Most significant differently expressed biological pathways (P < 0.05) examined with KEGG database in artificial insemination of P1 fraction (P1-AI) group in comparison with the Control group.

	*PATHWAY ID*	*PATHWAY NAME*	*ENRICHMENT SCORE*	*P-VALUE*	*GENES ALTERED%*	*GENE LIST*
**EndoCvx**						
*1*	KEGG_103	Steroid biosynthesis	6.1	0.002	12.2	*SQLE*, *MSMO1*, *CYP51*
*2*	KEGG_110	N-glycans biosynthesis	4	0.01	10.8	***ALG8***, ***B4GALT3***, ***DPM2***, ***UT8***
*3*	KEGG_8	Glycosaminoglycan biosynthesis	3.3	0.03	18.1	***B4GALT3***, ***FUT8***
**DistEndom**						
*1*	KEGG_91	Rap1 signaling pathway	5.6	0.003	7.3	*ARAP3*, *KIT*, ***PLCB4***, *FGF17*, *FGF9*, *FGFR1*, ***FLT4***, ***ITGAL***, ***SKAP1***, ***CALM1***
**ProxEndom**						
*1*	KEGG_103	Steroid biosynthesis	5.7	0.0002	20.7	***TM7SF2***, ***LIPA***, ***FAXDC2***, *CYP51*
*2*	KEGG_91	Rap1 signaling pathway	3.1	0.04	9.5	*FGFR1*, *KIT*, *ANGPT2*, ***FGFR2***, *SIPA1L2*, *GNAS*, ***ITGAL***, *PLCB1*, *IGF1* ***EFNA4***, *RAPGEF2*, ***LPAR3***, ***LPAR2***
*3*	KEGG_250	Glycosaminoglycan degradation	3	0.05	20	***GUSB***, ***HEXA***, ***NAGLU***
**UTJ**						
*1*	KEGG_91	Rap1 signaling pathway	6.5	0.001	12.4	***FIGF***, *ARAP2*, ***IGF1R*** *LPAR1*, *KIT*, ***RRAS***, ***INSR***, ***AKT3***, ***ITGAL***, ***MAP2K1***, ***PLCB4***, *TLN1*, *IGF1*, ***FGF1***, ***SRC***, *SKAP1*, *CTNND1*
*2*	KEGG_49	Adherens junction	5.7	0.003	17.4	***CSNK2A2***, ***IGF1R***, ***INSR***, ***SORBS1***, ***SRC***, ***WASF1***
*3*	KEGG_139	GnRH signaling pathway	3.3	0.03	12.3	***FSHB***, ***CAMK2G***, ***MAP2K1***, ***PLCB4***, *GNRHR2*, ***SRC***, ***MAP3K3***
*4*	KEGG_34	Focal adhesion	3.2	0.04	9.7	***FIGF***, ***LAMA4***, *VAV2*, ***IGF1R***, ***THBS3***, ***ITGA8***, ***AKT3***, ***PAK7***, ***MAP2K1***, ***TLN1***, *IGF1*, ***SRC***
**Isth**						
*1*	KEGG_110	N-glycans biosynthesis	4.2	0.01	21.6	*ALG8*, *B4GALT1*, *DAD1*, *DPM1*, *MAN2A1*, *MGAT2*, *MOGS*, *STT3A*
*2*	KEGG_252	mTOR signaling pathway	4.1	0.01	20	***AKT3***, *IGF1*, *MLST8*, ***PIK3CD***, ***PRKAA2***, *PRKCA*, ***RRAGC***, ***ULK1***, ***VEGFA***
**Inf**						
*1*	KEGG_66	FoxO signaling pathway	5.8	0.002	25.8	***BCL6***, ***BRAF***, *CCNB3*, ***CCNG2***, *CDKN2D*, ***EP300***, ***FOXO3***, *GADD45G*, *IGF1*, ***IGF1R***, ***INSR***, *IRS1*, ***MAPK10***, *MDM2*, *PCK2*, *PIK3R2*, *PLK3*, *PLK4*, *PRMT1*, ***SIRT1***, ***SOS2***, *STK11*, ***TGFBR1***, ***TNFSF10***
*2*	KEGG_59	Cell cycle	4	0.01	24.3	*BUB3*, *CCNB3*, ***CCNH***, *CDC45*, *CDK1,CDKN2D*, *E2F1*, *E2F4*, *E2F5*, ***EP300***, *ESPL1*, *GADD45G*, *MDM2*, *PTTG1*, *TP53*, *YWHAG*, *YWHAH*, *YWHAQ*
*3*	KEGG_110	N-glycans biosynthesis	3.4	0.03	24.5	***ALG13***, *DAD1*, *DDOST*, *DOLPP1*, *DPM3*, ***MAN1A1***, *MGAT4A*, ***MGAT5***, *MOGS*, *STT3A*
*4*	KEGG_195	HIF-1 signaling pathway	3	0.04	22.1	***ARNT***, ***CAMK2D***, ***EGLN1***, *EIF4E*, ***EP300***, *EPO*, *GAPDH*, ***IGF1***, ***IGF1R***, *INSR*, ***LTBR***, *NFKB1*, *PDHB*, *PDHB*, *PIK3R2*, *PLCG1*, ***TEK***, *VHL*

*Upregulated genes are marked in bold; the unmarked were downregulated.

Endocervix (EndoCvx), Endometrium (DistEndom, ProxEndom), and endosalpinx from Utero-tubal Junction (UTJ), Isthmus (Isth), Infundibulum (Inf).

**Table 6:** List of the most significant differently expressed biological pathways (P < 0.05) examined with KEGG database in artificial deposition of sperm-free seminal plasma (SP) from P1-fraction (SP-P1) group, in comparison with the Control group.

	*PATHWAY ID*	*PATHWAY NAME*	*ENRICHMENT SCORE*	*P-VALUE*	*GENES ALTERED%*	*GENE LIST*
**EndoCvx**						
*1*	KEGG_105	Cell adhesion molecules	3.7	0.02	5.5	***CD8B***, ***SLA-1***, *SLA-DOA*, *SLA-DQB1*, *VCAM1*
*2*	KEGG_110	N-glycans biosynthesis	3.5	0.03	8.1	***ALG13***, *ALG5*, ***B4GALT3***
**DistEndom**						
*1*	KEGG_133	Mismatch repair	4.9	0.007	31.2	*AKT2*, ***ATM***, *BCL2L1*, *CSF2RB*, *IRAK1*, *MYD88*, ***PPP3CB***, *RELA*, *RIPK1*, *TNFRSF1A*
*2*	KEGG_56	Apoptosis	4.7	0.082	19.2	*LIG1*, ***MSH2***, ***MSH3***, ***PMS2***, ***SSBP1***
**ProxEndom**						
*1*	KEGG_213	PI3K-Akt signaling pathway	5.6	0.003	8	*ANGPT1*, *ANGPT2*, ***CDK6***, *COL1A2*, *COL3A1*, *COL5A2*, *COL5A3*, *COL6A3*, ***EPO***, *FLT1*, ***IFN-ALPHA-9***, *ITGA5*,, *LAMA4*, ***LPAR3***, *PGF*, ***PIK3R5***, *PPP2CB*, *PRLR*
*2*	KEGG_34	Focal adhesion	3.6	0.02	8.1	*COL1A2*, *COL3A1*, *COL5A2*, *COL5A3*, *COL6A3*, *FLT1*, *ITGA5*, *LAMA4*, *PGF*, ***PIK3R5***
*3*	KEGG_195	HIF-1 signaling pathway	3.4	0.03	9.1	***EPO***, ***NPPA***, ***PIK3R5***, ***TFRC***
**UTJ**						
*1*	KEGG_139	GnRH signaling pathway	5.1	0.006	8.8	*ATF4*, ***GNRHR2***, *GRB2*, ***MAP2K1***, ***SOS2***
*2*	KEGG_46	Estrogen signaling pathway	4.8	0.008	8.1	ATF4, GRB2, **MAP2K1**, SHC1, **SOS2**
**Isth**						
*1*	KEGG_34	Focal adhesion	5.6	0.003	12.2	***AKT3***, ***BRAF***, ***FYN***, ***IBSP***, ***ITGA2B***, ***ITGA4***, *JUN*, ***LAMA4***, *MRLC2*, ***MYLK***, *MYLK2*, ***PTEN***, *SHC4*, ***SOS2***, ***VCL***
*2*	KEGG_98	Rap1 signaling pathway	5.5	0.004	11.7	***AKT3***, ***ARAP3***, ***BRAF***, ***CNR1***, *FGF17*, *FGF9*, ***GNAO1***, ***GNAQ*** ***INSR***, ***ITGA2B***, ***KRIT1***, ***LCP2***, ***P2RY1***, ***RAPGEF6***, ***RASGRP3***, *RGS14*
*3*	KEGG_66	FoxO signaling pathway	5.1	0.005	12.9	***AKT3***, ***ATM***, ***BRAF***, *CDKN1A*, *CDKN2B*, ***FOXO3***, ***INSR***, ***PRKAA2***, ***PTEN***, ***SIRT1***, ***SMAD4***, ***SOS2***
*4*	KEGG_213	PI3K-Akt signaling pathway	4.9	0.007	9.8	***AKT3***, *CDKN1A*, ***CRTC2***, *FGF17*, *FGF9*, ***FOXO3***, ***GHR***, *GNG13*, ***IBSP***, ***IL2RG***, ***INSR***, ***ITGA2B***, ***ITGA4***, ***JAK2***, ***LAMA4***, ***OSM***, ***PRKAA2***, ***PTEN***, ***SOS2***, *SYK*, *TP53*, *YWHAQ*
*5*	KEGG_46	Estrogen signaling pathway	3.9	0.02	13.1	***AKT3***, *FKBP4*, ***GNAO1***, ***GNAQ***, *JUN*, ***MMP9***, *SHC4*, ***SOS2***
*6*	KEGG_59	Cell cycle	3.8	0.02	12.2	***ATM***, ***CCNH***, *CDKN1A*, *CDKN2B*, ***RB1***, ***SMAD4***, ***STAG2***, *TP53*, *YWHAQ*
*7*	KEGG_252	mTOR signaling pathway	3.2	0.03	13.3	***AKT3***, ***BRAF***, ***PRKAA2***, ***PTEN***, ***RICTOR***, ***RRAGC***
*8*	KEGG_49	Adherens junction	3.1	0.04	13	***FYN***, ***INSR***, ***SMAD4***, ***VCL***, ***WASF1***, ***WASL***
**Inf**						
*1*	KEGG_195	HIF-1 signaling pathway	4.2	0.01	15.6	***ANGPT4***, *EGLN2*, *ENO3*, *GAPDH*, ***IFNGR1***, *IL-6*, *LTBR*, *MTOR*, *PIK3R2*, *PLCG1*, *RELA*, ***TEK***

*Upregulated genes are marked in bold; the unmarked were downregulated.

Endocervix (EndoCvx), Endometrium (DistEndom, ProxEndom), and endosalpinx from Utero-tubal Junction (UTJ), Isthmus (Isth) and Infundibulum (Inf).

**Table 7:** List of the most significant differently expressed biological pathways (P < 0.05) examined with KEGG database in artificial deposition of sperm-free seminal plasma (SP) from entire ejaculate (SP-Ejac) group, in comparison with the Control group.

	*PATHWAY ID*	*PATHWAY NAME*	*ENRICHMENT SCORE*	*P-VALUE*	*GENES ALTERED%*	*GENE LIST*
**EndoCvx**						
*1*	KEGG_105	Glycosaminoglycan biosynthesis	3.5	0.03	20	*CHST3*, *CHST7*, *DSE*
**DistEndom**						
*1*	KEGG_66	FoxO signaling pathway	4.1	0.01	10.8	***ATG12***, ***ATM***, *BCL6*, *CDKN2D*, *G6PC2*, *HOMER2*, ***KRAS***, ***MAPK10***, ***PRKAB1***, ***PRKAB2***
*2*	KEGG_59	Cell cycle	3.5	0.02	10.8	***ANAPC13***, ***ANAPC5***, ***ATM***, *CDKN2D*, *E2F3*, ***HDAC2***, ***SMC3***, ***STAG2***
*3*	KEGG_105	Glycosaminoglycan biosynthesis	3.3	0.03	20	*CHST7*, *CHST12*, *CHST15*
**ProxEndom**						
*1*	KEGG_213	PI3K-Akt signaling pathway	3.6	0.02	5.6	*COL5A3*, *CSF3*, *FGFR1*, *IFN-ALPHA-9*, *JAK1*, *MCL1*, *OSM*, ***PKN3***, ***PPP2R5D***, *PRLR*, *TN-X*, *TSC2*

*Upregulated genes are marked in bold; the unmarked were downregulated.

Endocervix (EndoCvx), Endometrium (DistEndom, ProxEndom).

## Discussion

Although the effects of semen and SP on the porcine uterus have been previously characterized^13^, the present study provides, to the best of our knowledge, the first description of a genome-wide gene expression analysis of specific endo-cervical, endometrial and endosalpingeal responses to semen or SP (either of the entire ejaculate or of the sperm-peak fraction of the boar ejaculate). We have compared the portion of the ejaculate first contacting the internal genital tract of the sow^5,14^ with the non-exposed control sows in the same period of the oestrous cycle: peri-ovulation. Considering the transcriptomic effects we detected during the pre-ovulatory oestrus can continue over ovulation and even be visible during the progesterone-dominated stages of the oestrus cycle/early pregnancy, a follow-up study should include experimental stimulation during the post-ovulatory oestrus.

Differences were seen among the various exposures (sperm-containing or solely seminal plasma). Despite these differences were clear, we should not forget that there are other factors that could play roles in the effects, and preliminarily appear as confounding factors. For instance, the entry into oestrus, and the use of manual detection of oestrus behavior both cause elevations of female oxytocin in peripheral blood^15^. Mating dramatically increases this oxytocin release^16^ while manipulation of uterus and its cervix causes intermediate responses^17^. The study, however, was focused on those aspects solely vinculated to the entry and interaction of semen on/with the female internal genital tract. We separated the whole ejaculate (natural mating) from AI with the sperm-peak fraction in a fixed volume, the 1st 10 mL of the sperm-rich fraction (SRF) hereby defined as P1, which contains 25% of the total number of spermatozoa of the entire ejaculate^5,18,19^. Using such fractionation, we were able to use the same volumen and relative sperm concentration from P1 (containing spermatozoa), derived from ejaculates of 2 boar siblings, whose sperm quality was very similar in between.

The retrieval of mucosal samples 24 hours after semen or SP-only exposure explores the period between semen deposition and the colonization of the sperm reservoir at the UTJ, including sperm transport towards the oviduct. The enrichment analysis of the collected mucosae revealed an overrepresentation of several genes and pathways associated with many reproductive-related processes relevant for the colonization of the sperm reservoirs such as: sperm transport and survival, antioxidant activity, sperm binding and steroid biosynthesis, amongst others.

The phosphatidylinositol 3-kinase (PI3K-Akt) signaling pathway, was one of the most significant altered pathways found in the endometria (Dist and Prox) as well as in the ampulla after mating. This pathway was also altered after SP-Ejac (ProxEndom) and after SP-P1 (ProxEndom and Isth). The PI3K-Akt signaling pathway plays a pivotal role in numerous reproductive processes as an important regulator of cell proliferation and survival, being widely studied due to its participation in endometrial receptivity in several species including the pig^20,21^. Although the PI3K-Akt signaling pathway is considered strongly associated to uterine decidualization in invasive-implantation species, there is evidence that in porcine (a non-invasive-implantation species), the endometrium undergoes a continuous cycle of changes during pregnancy, including events such as cellular remodeling as well as maintaining hormonal responsiveness and secretory function by the uterine epithelium^22^. These processes prepare the genital tract mucosae for fertilization, embryo development and implantation^23^. Interestingly, we found 39 altered transcripts related to the PI3K-Akt signaling pathway in the endometrial samples within the Mating group, suggesting that just 24 hours after semen deposition, the mechanisms that would lead to building up a receptive endometrial environment for embryo survival and implantation are already activated. One of the most interesting genes associated with this pathway is the colony stimulation factor 1 and its receptor (the *CSF1-CSF1R* complex), that were up-regulated in the endometrial, ampullar and infundibular mucosae after mating. The *CSF1-CSF1R* complex has been widely related to many biological processes, such as protein phosphorylation, cell proliferation, cell migration, cellular response to macrophage colony-stimulating factor stimulus, cell-cell junction maintenance and cellular response to cytokine stimulus^24^. Here, we provide evidence that this complex is overexpressed within the entire female reproductive tract after mating, thus triggering the activation of several cellular processes.

Additionally, the FoxO signaling pathway, regulated by the PI3K-Akt signaling pathway^25,26^, is overrepresented in the upper segments of the oviduct (ampulla and infundibulum) mostly by upregulation. The FoxO transcription factors regulate the expression of genes that are involved in apoptosis, oxidative stress resistance, DNA repair, cell cycle transitions and differentiation^27,28^ and play important roles in reproductive processes by increasing cellular antioxidant activity^29–31^. All of these processes are relevant to the site of fertilization, with the presence of immunologically foreign-spermatozoa (as well as a high rate of epithelial cell turnover^32^). Specially, *FOXO3* and *FOXO4*, which were up-regulated in our experiment by Mating or AI with the P1-semen fraction, have been reported to provide cell protection against ROS-induced damage by directly increasing their quantities of manganese superoxide dismutase (MnSOD)^33,34^. Insemination triggers an inflammatory cascade in the internal female genital tract during the peri-ovulatory period^6,35^; an inflammatory process intimately related to the action of reactive oxygen species (ROS)^36^. Spermatozoa in general, but those of boar in particular, are highly vulnerable to oxidative stress due to the high polyunsaturated acid content in their membranes^37^. Therefore, we hypothesize that FoxOs are physiologically activated in the oviduct in response to semen exposure in order to overcome the negative impact of oxygen-induced damage surrounding the inflammatory cascade. Once the spermatozoa reach the oviduct, they elicit changes in gene expression to promote a favorable environment by influencing the release of many oviduct secretions^38^. We identified several genes encoding oviduct-specific proteins with important roles in sperm binding, sperm protection, smooth muscle contractility or gamete-oviduct interactions, suggesting that entry of semen leads to the activation of oviduct mechanisms that trigger these processes. There are several reports studying the mechanisms that might be responsible for retaining spermatozoa in the oviductal reservoir of the sow^38^, although the molecular interactions that influence this phenomenon as yet unclear. Considerable novel evidence supports the involvement of glycans in oviductal sperm colonization^39^ which includes the role played N- and O-linked glycans found in oviduct epithelial cells that, by binding specific receptors found in the sperm head membrane^40^, aid establishing the tubal reservoir in the pig^41^. Sperm binding to oviduct cells via glycans have been even proposed as additional biomarkers for semen analysis^42^. At 24 hours after semen deposition, *ST3GAL5*, *MAN1C1*, *B4GALT1* and *MAN2A1*, which encode enzymes that modify already syntesized N-linked and/or O-linked glycans by adding or removing a single monosaccharide, were upregulated in the Isthmic segment after Mating and P1-AI treatments respectively. Such findings support the prevailing concept that pig spermatozoa, being stored for up to 30–36 hours in the oviductal reservoir, maintain viability and fertilizing capacity until ovulation takes place^12,43,44^. In addition, various carbohydrate sulfotransferases genes (*CHST7*, *CHST3* and *CHST12*), involved in the catalysis of the sulfation of glycosaminoglycans (GAGs)^45^ were also overrepresented in our study. The non-sulfated GAG hyaluronan is produced by oviduct epithelial cells to act multipurposely, being related to the capture of spermatozoa during their storage in the isthmus reservoir by adhering to sperm surface membrane, to sperm capacitation, acrosome reaction and fertilization^32,38,46,47^. We identified mostly downregulated targets implicated in the synthesis of HA or its receptors in the Infundibular segment; *HAS2*, *HYAL2*, *VCAN*, *HMMR*, *SPAM1* after Mating, and of *VCAN*, *HAPLN1*, *HABP4* in the P1-AI group. Similarly, we observed many genes encoding binding-molecules such as annexin were either up or downregulated in our study. Ignotz *et al*.^48^ reported in the cow, that oviductal annexin could act as possible oviduct receptors for sperm surface proteins and thus serve to hold the spermatozoa in the oviductal reservoir during the pre-ovulatory period. In the pig, annexins have also been isolated from oviductal epithelial cells probably also involved in sperm-oviduct interactions by acting as sperm-binding proteins^49^. In our study, *ANXA5*, a major constituent of the members of the annexin family, was downregulated after Mating. In addition, we found upregulated genes that encode proteins that have heparin affinity such as *PRELP*, *LPL*, *POSTN*, *ABI3BP*, *FGF10* after Mating; and *PTN*, *PRELP*, *VEGFA* after P1-AI exposure. There is evidence that sulfated GAGs, as heparin or heparan-sulfate, produced by the oviductal epithelium^50^ are powerful inhibitors of sperm binding to oviductal cells^47^ and so, concerted interplay between various GAGs can be involved when sperm release from the pig oviductal reservoir occurs^43,44^.

What role can then the seminal plasma play? The capacity of porcine testicular spermatozoa to bind the oviduct epithelium can be advanced by components of the fluid of the cauda epididymides^51^, i.e. the fluid prevailing in the P1-fraction. Seminal plasma spermadhesins are also related to sperm binding to the epithelium^52^ act prior to/during capacitation^53,54^, during the sperm release from the oviductal reservoir^55^ and even to fertilization, particularly during binding to the zona-pellucida^56^, commonly acting via their heparin-binding domain^57,58^. The fact that we have found some altered transcripts associated with either sperm binding or detachment from the oviductal reservoir is consistent with the general concept that the sperm release from oviductal epithelium is a gradual process that restrict the number of sperm in the site of fertilization to control polyspermy^59^ and maintains sperm in a protective state until they are fertilization-competent^18^, so it seems plausible to find active mechanisms of either attach or detachment during the peri-ovulatory period, including the action of progesterone and CatSper^60^ during sperm capacitation, illustrated by the experimental blockade of sperm release inhibition caused by inhibition of the progesterone receptor that activates CatSper^61^. The reason why these results were not so evident in the P1-AI group may be related to the number of spermatozoa deposited into the reproductive tract, which is approximately 30 times lower than those during natural mating^62^. In addition, another mechanism that has been implicated in sperm detachment from the oviductal reservoir is the contractility of the myosalpinx smooth muscle^63^. There is extensive evidence that concerted contractions of the myometrium and the myosalpinx are probably primarily responsible for moving sperm and oocytes to the fertilization point^1,18,63^. In this context, we found many downregulated genes encoding cAMP-binding proteins which are the major responsible of inhibiting smooth muscle contractility to maintain uterine quiescence during pregnancy^64^. In the smooth muscle cells of the myometrium or myosalpinx, cAMP functions as a second messenger that activates protein kinase-A (PKA)^65^, a multifunctional kinase that phosphorylates downstream targets to suppresses contractility by decreasing the intracellular concentration of free Ca^2+^ and inhibiting myosin light chain kinase activity^66,67^. Interestingly, regulatory subunits of protein Kinase A, such as *PRKAR1A*, were downregulated in the Ampulla and Infundibulum in the M and P1-AI groups, and *PRKAR2A* was downregulated in Ampulla after Mating and in the Infundibulum in both M and P1-AI groups.

Moreover, cyclic nucleotide phosphodiesterases (PDEs), which are degradation enzymes known for their role in cAMP breakdown, were mainly upregulated. Specifically, *PDE-*4 isoforms were detected. These have been widely studied in several species, and catalyze the hydrolysis and inactivation of cAMP^68,69^, lowering its concentration in the smooth muscle cells. Based on our findings, we can clearly hypothesize that the presence of spermatozoa in the oviduct could be influencing myosalpinx motility. These effects were more obvious after Mating (*PDE-4* up-regulated in Isthmus, Ampulla and Infundibulum) followed by P1-AI (*PDE-4* up-regulated in Infundibulum) and SPP1 (*PDE-4* up-regulated in Isthmus). These differences could be explained by the fact that when natural mating takes place, the presence of a boar (separate from insemination itself) induces central oxytocin release and thus increases smooth muscle motility in the sow^16^.

Of note, the SP groups showed the lowest changes in terms of overall transcriptional modifications. In the pig, as in other mammals, there is little likelihood that the SP reaches the oviduct after semen deposition because the utero-tubal junction acts as a physiological barrier^38^. Therefore, the evidence presented in this report supports the hypothesis that spermatozoa themselves and not the SP are the major agent responsible for the molecular changes elucidated in the pig oviduct.

However, SP molecules can support sperm survival along the female genital tract and thus, enhance reproductive success^70^. In addition, previous studies suggest that seminal estrogen has a positive effect on sperm transport and fertilization and is also responsible for rapid sperm elimination from the uterus through the cervix in the pig^71^. It has also been reported that sperm binding to the oviductal cells is triggered by high concentrations of estrogen within the oviduct^72,73^. Interestingly, the SP-P1 group showed an overrepresentation of the Estrogen signaling pathway in our study. Of particular interest was the up-regulation of *HSD17B8* in the UTJ, suggesting that the SP may play a role in sperm attachment and release from the oviduct reservoir via estrogen signaling regulation. It is known that exosomes present in SP bind to the sperm head membrane and improve membrane integrity^74^. Also, it has been stated that proteins present in SP are involved in the immune and inflammatory response in the uterus^75^. Moreover, there is evidence that mRNAs and miRNAs are heavily involved in boar sperm response to environmental stimuli, apoptosis, and metabolic activities^76^. However, the components of SP responsible of the alterations found in this study are yet to be determined.

In conclusion, our study underlies that boar spermatozoa and/or SP have the ability to modify molecular patterns in the endocervix, endometrium and endosalpinx during the peri-ovulatory period, supporting the idea that these contribute to the development of a receptive environment to prepare for successful fertilization, as well as for an adequate milieu for zygote/early embryo development.

## Materials and Methods

### Ethics statement of the interventional study

All animal husbandry and experimental handling was performed in compliance with the European Community (Directive 2010/63/EU) and current Swedish legislation (SJVFS 2017:40), following the Reduction principles of the 3Rs on animal experimentation (Replacement, Reduction and Refinement) while maintaining enough numbers of biological replicates from distinct animals, to obtain sufficiently reliable enough estimates of variation among samples within conditions to distinguish true differences between conditions. This applied to both treatment and control conditions. The experiments were approved in advance by the “Regional Committee for Ethical Approval of Animal Experiments” (Linköpings Djurförsöksetiska nämnd) in Linköping, Sweden (permits no. 75–12 and no. ID1400).

### Experimental design

Changes in the general transcriptome profile of the pre/peri-ovulatory porcine endocervix, endometrium (distal and proximal) and endosalpinx (UTJ, isthmus, ampulla and infundibulum), were studied 24 h after the deposition of semen (i.e. spermatozoa and SP) or of sperm-free SP. Twenty sows displaying standing oestrus in the presence of a boar were equally and at-random allotted to a Control group (n = 4), where females were cervically infused with 50 mL of the protein-free extender “Beltsville Thawing Solution” (BTS)^77^ or a series of treatment groups; including either entry of semen (spermatozoa and the accompanying SP) either by mating (M, whole ejaculate) or AI of the sperm-peak fraction e.g. the first 10 mL of the sperm-rich fraction (P1) (P1-AI, contained 25% of the total sperm numbers of the ejaculates of the boars used which were ~40 billion spermatozoa) or counterparts holding solely the sperm-free SP (entire SP; SP-Ejac) or SP from the P1 fraction (SP-P1). For the M group (n = 4), the sows were each mated with a single male; in the P1-AI (n = 4) females were cervically artificially inseminated with the ejaculated P1-sperm-peak fraction (extended to 50 mL with BTS) while for SP-Ejac (n = 4) sows were cervically infused with sperm-free SP of the entire ejaculate (50 mL) and finally, in the SP-P1 (n = 4), females were cervically infused with sperm-free SP harvested from ejaculated P1-fractions (pool, 50 mL). Samples from endocervix (EndoCvx), endometrium (distal: DistEndom or proximal: ProxEndom) and endosalpinx from 4 different segments; the utero-tubal junction (UTJ), isthmus (Isth), ampulla (Amp) and infundibulum (Inf) were surgically retrieved under general anesthesia, 24 h after the interventions (Control/Treatments).

### Animal management

Pigs of Swedish Landrace breed were recruited from a controlled breeding farm, as weaned sows (parity 1–3, n = 20) and young mature boars (9–11 months, n = 5) of good semen quality (<100 mL volume, >60 × 10^9^ total sperm number, >70% sperm motility, and >75% morphologically normal-looking spermatozoa, controlled weekly)^6^. Throughout all experiments, animals were handled carefully to avoid any unnecessary stress. The animals were individually kept in separate pens at the Translational Medicine Centre (TMC/CBR-3) of Linköping University under controlled temperature and light regimes (12 h:12 h light/dark cycle). Pigs were fed with commercial feedstuff (Lantmännen, Stockholm, Sweden) according to national standards^78^, provided with water *ad libitum* and receiving the same management.

### Semen collection and SP harvesting

Ejaculates, collected using the gloved-hand method, and with at least 70% motile and 75% morphologically normal-looking spermatozoa immediately after collection, were used for the experiments. The SP was harvested either from the whole ejaculate or from the sperm-peak P1-fraction after double centrifugation at 1,500 × g for 10 min, and microscopically checked for presence of spermatozoa. The harvested sperm-free crude SP was stored at −20 °C until used.

### Handling of sows

Detection of estrus was performed during snout-to-snout contact of females with the presence of mature boars while applying back-pressure to test for standing estrus reflex twice daily, beginning one day after weaning. When sows showed standing estrus reflex they were used in the experiments, considered to be on the first day of behavioral estrus. Sows were cervically inseminated/infused using disposable conventional AI-catheters (Minitüb, Munich, Germany).

### Collection of genital tract samples

All sows were subjected to mid-ventral laparotomies to collect the tissue samples 24 h after mating/inseminations (pre-/peri-ovulation period), as described by Alvarez-Rodriguez^6^. Briefly, sows were sedated by the i.m. administration of a mixture of 5 mg dexmeditomedine (Dexdomitor, Orion Pharma Animal Health, Sollentuna, Sweden) and 100 mg tiletamine hydrochloride/zolazepam hydrochloride (Zoletil vet, Virbac A/S, Kolding, Denmark) followed by anaesthesia induced i.v. with sodium thiopental (Abbot Scandinavia AB, Solna, Sweden, 7 mg/kg bw), and maintained with isoflurane (3.5–5%, Baxter Medical AB, Kista, Sweden) administered via a tracheal cuffed tube by a close-circuit PVC-ventilator (Servo ventilator 900 D, SIEMENS-ELEMA AB, Solna, Sweden). The number of pre-ovulatory follicles or eventual new ovulations per sow was 22.30 ± 7.29 (mean +− standard deviation) pre-ovulatory follicles, without significant differences between sow-groups. Peripheral blood was collected for estradiol 17ß (E_2_) and progesterone ELISA analyses in plasma, which confirmed the animals were in pre-/peri-ovulatory estrus (mean ± SD pg/mL for P4 = 0.77 ± 0.35 in all sows; and for E_2_ ranging 376.50 ± 27.76–294.20 ± 80.24 in all experimental groups, e.g., without significant differences). Segments of the female genital tract segments were immediately retrieved and stored at –80 °C in RNAlater (Ambion, Thermo Fisher Scientific Baltics UAB, Vilnius, Lithuania) until analysed.

### Transcriptome analysis

Analyses of the tissue samples was done as described by Alvarez-Rodriguez^6^. In brief, total RNA was isolated from tissue samples using Trizol reagent (Invitrogen, Carlsbad, CA, USA) and quality assessment was performed using an Agilent 2100 Bioanalyzer (Agilent Technologies, Santa Clara, CA, USA) according to the manufacturer’s instructions. The RNA integrity number (RIN) values obtained were in the range of 8 to 10, which guarantied the homogeneity and high quality of the samples. Equal amounts of total RNA (250 ng) from each sample were used to make cDNA using GeneChip® Whole Transcript Plus reagent kit (Affymetrix, Santa Clara, CA, USA) following the manufacturer protocol. cDNA was then hybridized and loaded on the array chip (GeneChip® Porcine Gene 1.0 ST Array, Affymetrix Inc., 3420 Central Expressway, Santa Clara, CA 95051, USA), incubated at 45 °C under 60 rotations per min, for 16 h. The hybridized cartridge array chip was then unloaded and subjected to washing and staining using a GeneChip® Fluidics Station 450 (Affymetrix), to be finally scanned using the Affymetrix GeneChip® scanner GCS3000.

### Analysis of microarray data and bioinformatics

The intensity data of each array chip was processed using the robust multi-array average (RMA) normalization, computing average intensity values by background adjustment, quantile normalization among arrays and finally log2 transformation for extracting the expression values of each transcript in the probe set, as implemented in the official Transcriptome Analysis Console (TAC; version 4.0) from Affymetrix. The statistical analysis of the normalized gene expression data was performed using a linear model using the empirical Bayes’ approach as implemented in the package “limma” was used to calculate differentially expressed transcripts using a Benjamini-Hochberg (q < 0.05) correction to control for multiple testing to control type-I errors^79^ and a fold change (FC) > 1 or <−1.

### Enrichment analysis

Data was then analyzed using the PANTHER Classification System for gene ontology (GO) terms^80^. The differentially expressed genes (p < 0.05) were screened for additional molecular functions with the protein knowledge base of the UniProt Consortium^81^ to confirm they were correctly classified as different groups. An additional analysis of altered pathways was performed using Partek Genomics Suite 7.0 (Partek) using the KEGG database^82^. Graphical illustration of overrepresented GO terms and pathways was produced with the Cytoscape v3.0.0 application ClueGO v2.0.3^83^.

## Supplementary information

Supplementary information.
