# Supplementary material for: The role of semen and seminal plasma in inducing large-scale genomic changes in the female porcine peri-ovulatory tract
Source: Sci Rep. 2020 Mar 19;10:5061. doi: 10.1038/s41598-020-60810-z (PMC7081221; doi:10.1038/s41598-020-60810-z)

# Supplementary Figure 1-14.

**Differential expression (up- and down-regulation) of annotated genes** in the mucosa of the internal genital tract of sows after Mating or artificial deposition of the sperm-peak portion P1 (P1-AI); sperm-free seminal plasma of the whole ejaculate (SP-Ejac) or of pooled P1 (SP-P1), **classified in molecular function (Fig 1 to 7) and biological function (Fig 8 to 14).** All treatments were compared to Control (AI with 50 mL of BTS). The numbers represent the number of differentially expressed genes ($p$-value $< 0.05$). Endocervix (EndoCvx), endometrium (distal: DistEndom or proximal: ProxEndom) and endosalpinx from 4 different segments; the utero-tubal junction (UTJ), isthmus (Isth), ampulla (Amp) and infundibulum (Inf).

**Supplementary Figure 1.** Molecular function in EndoCvx

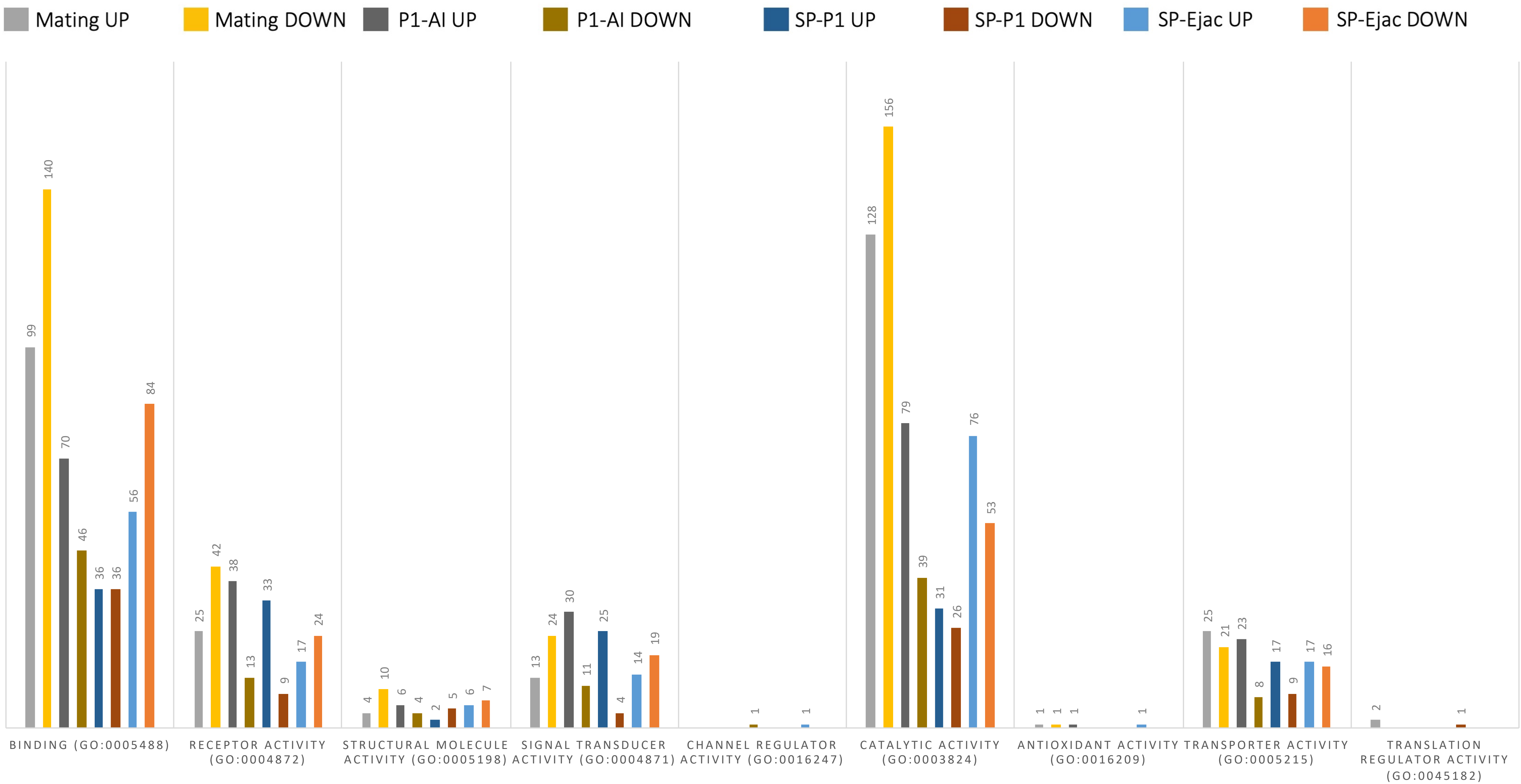

**Supplementary Figure 2.** Molecular function in DistEndom

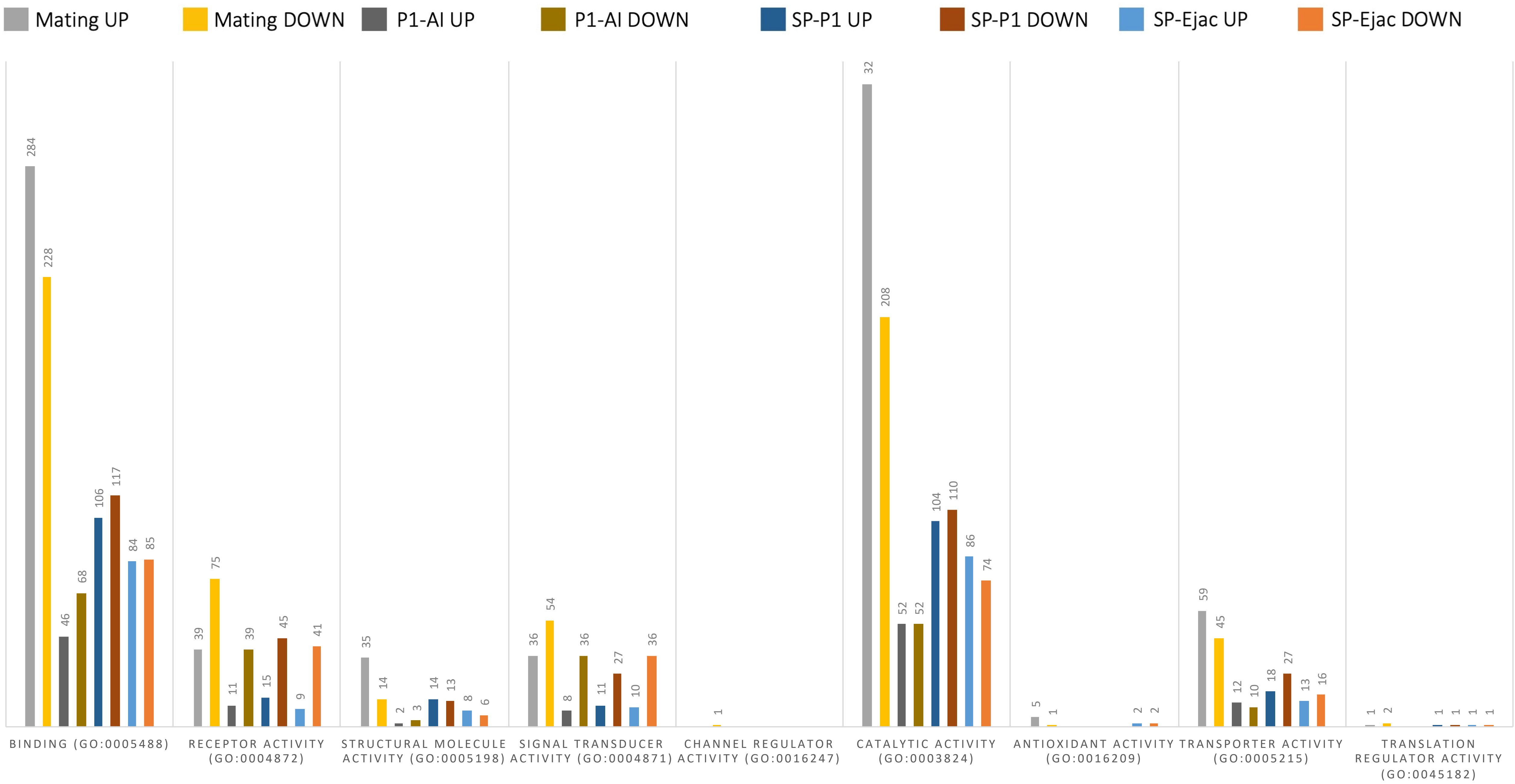

**Supplementary Figure 3.** Molecular function in ProxEndom

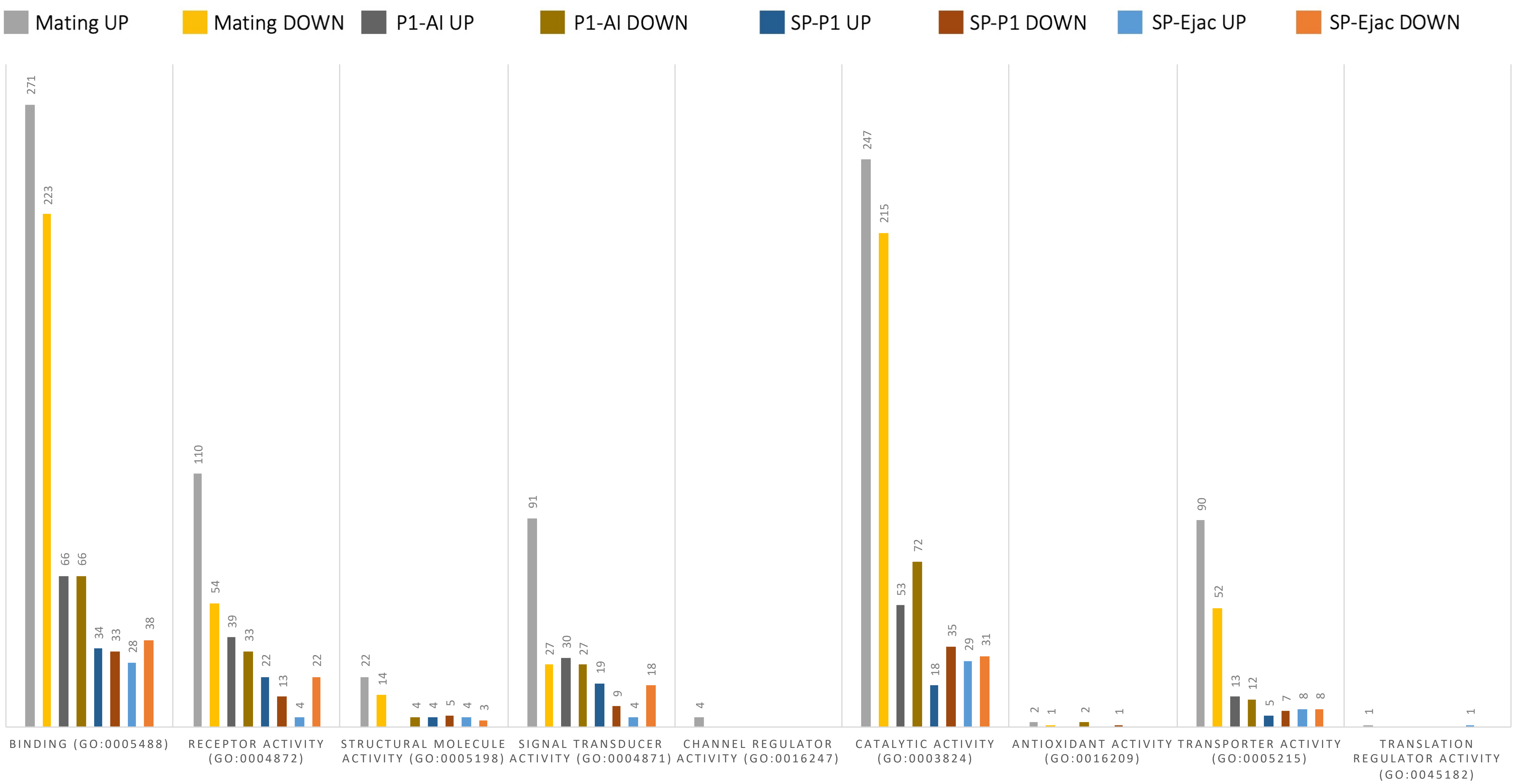

**Supplementary Figure 4.** Molecular function in UTJ

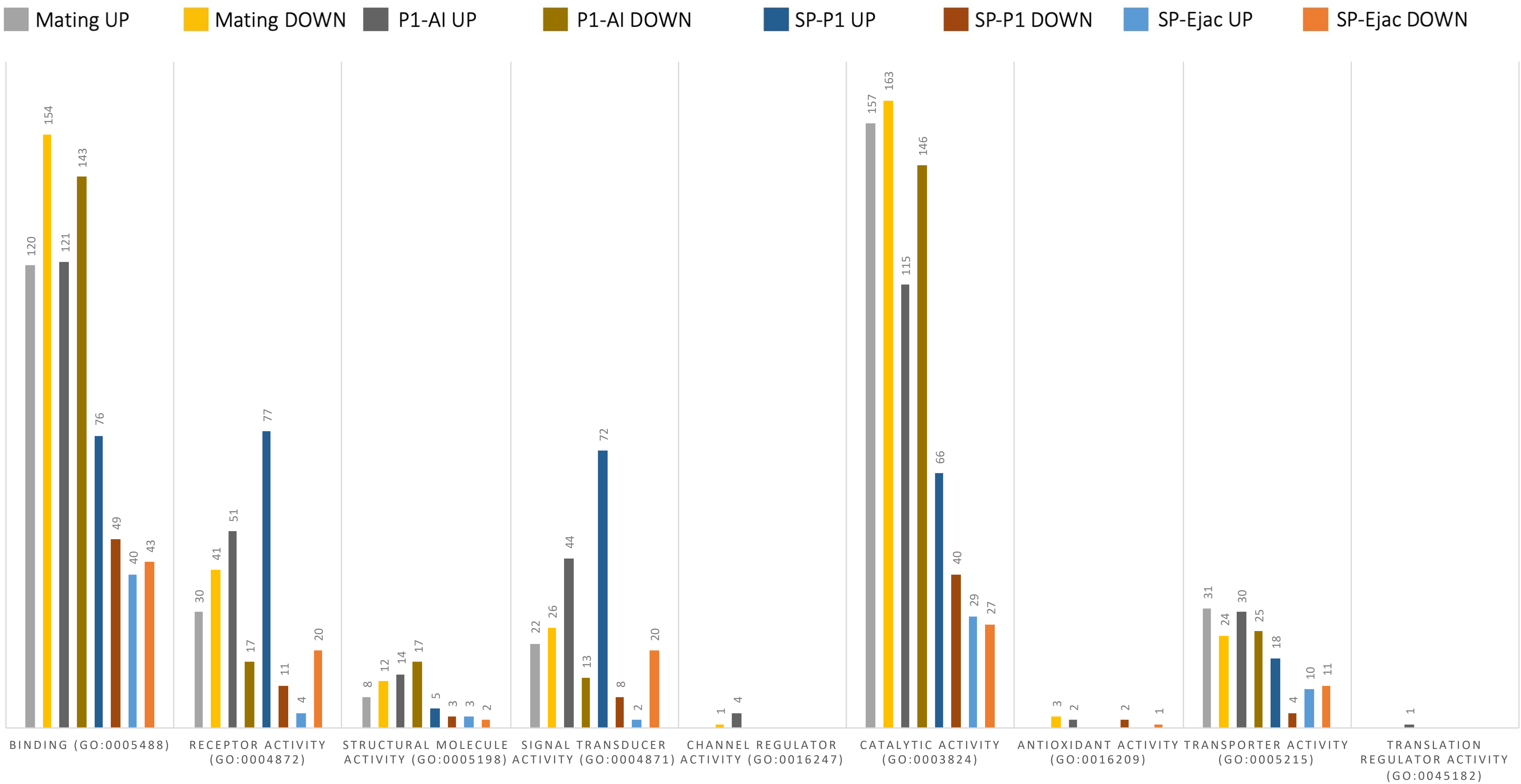

**Supplementary Figure 5.** Molecular function in Isth

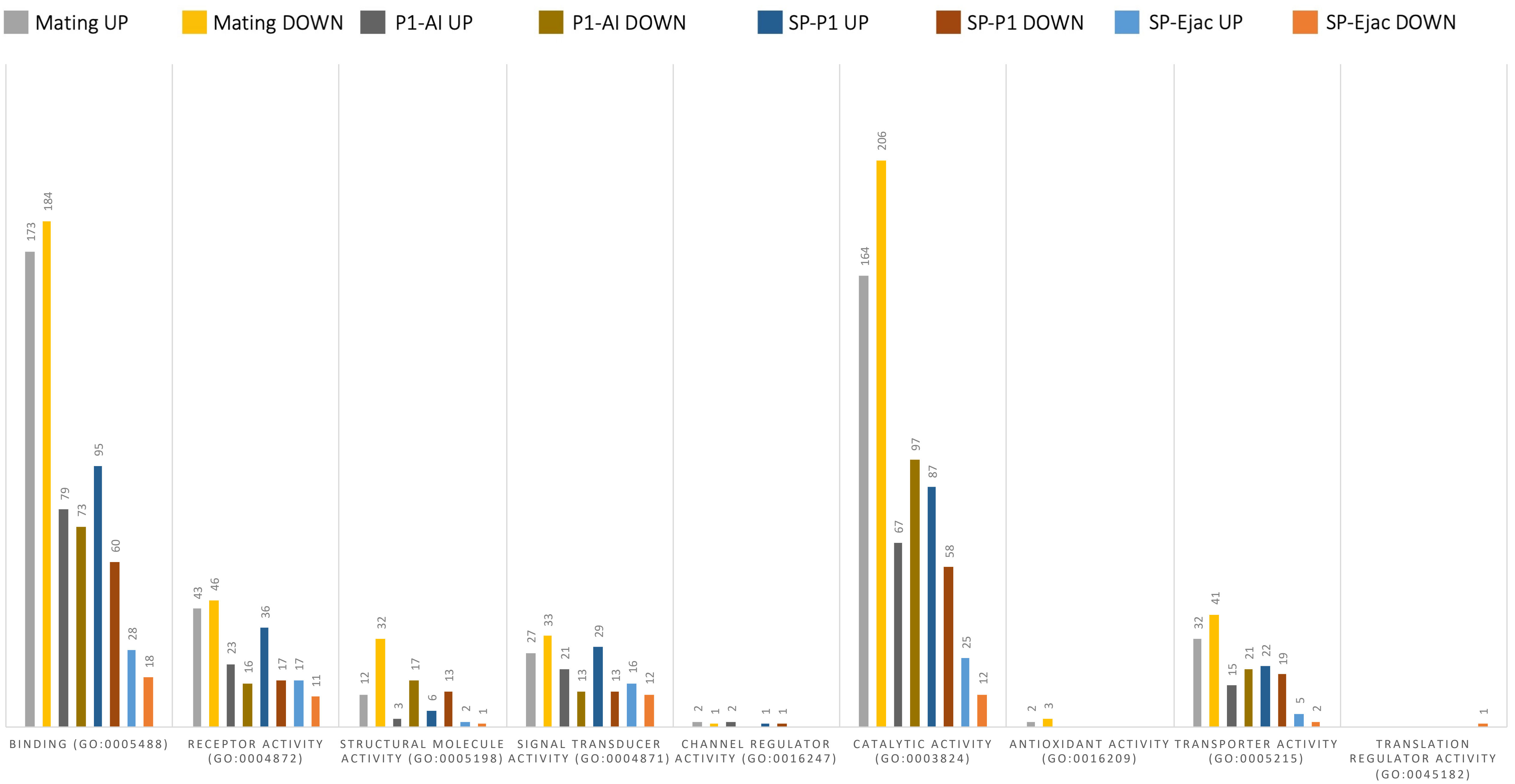

**Supplementary Figure 6.** Molecular function in Amp

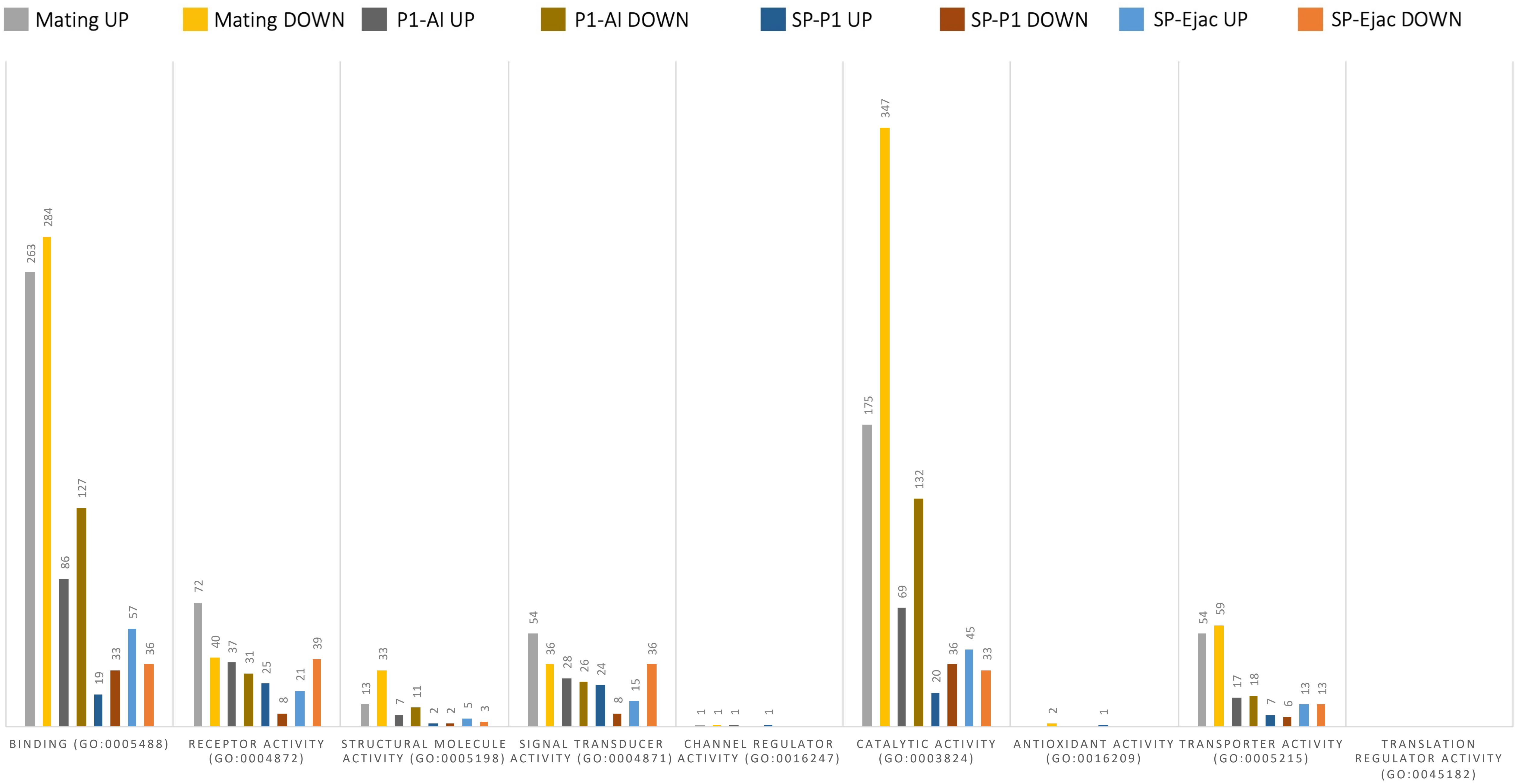

**Supplementary Figure 7.** Molecular function in Inf

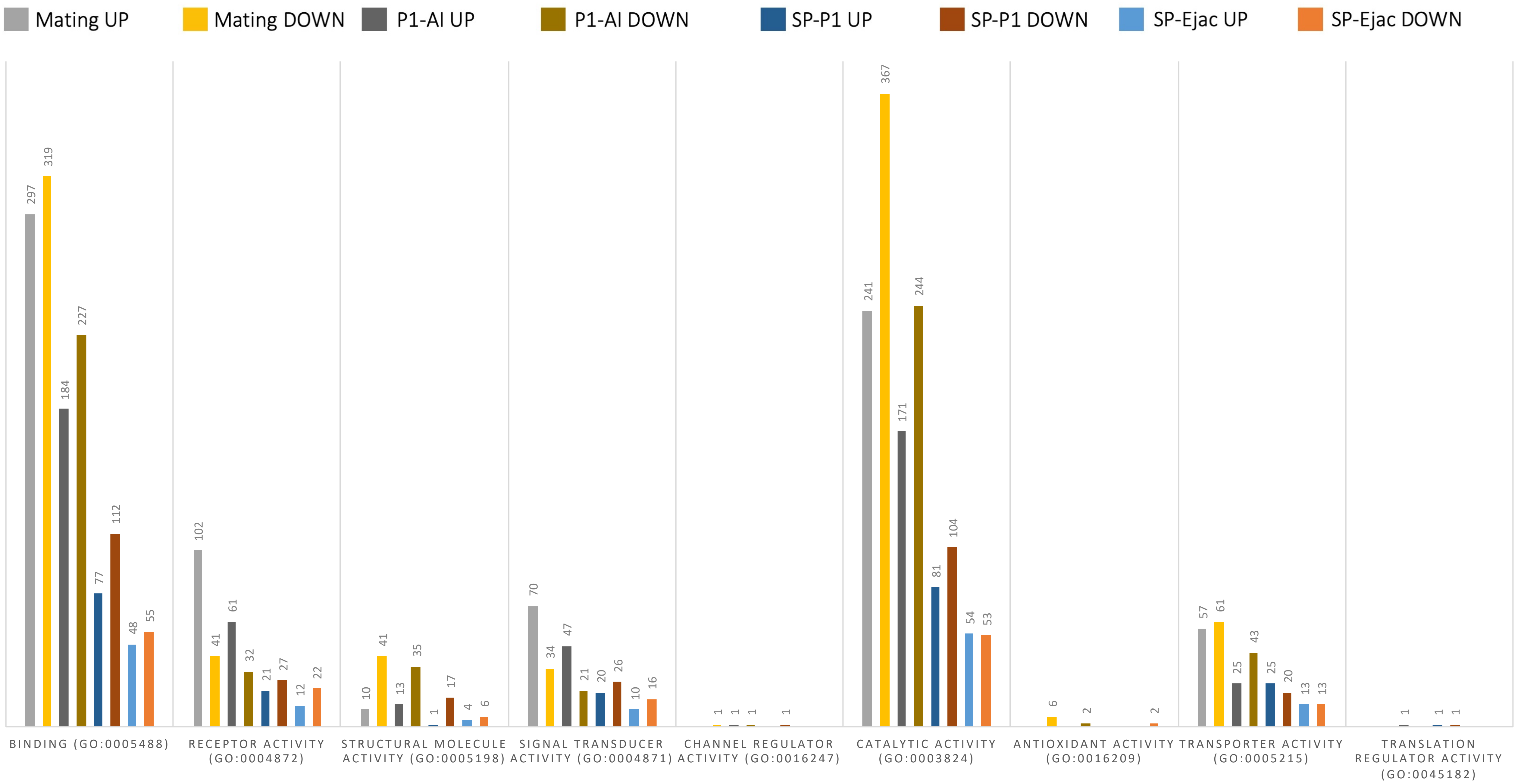

**Supplementary Figure 8.** Biological process in EndoCvx

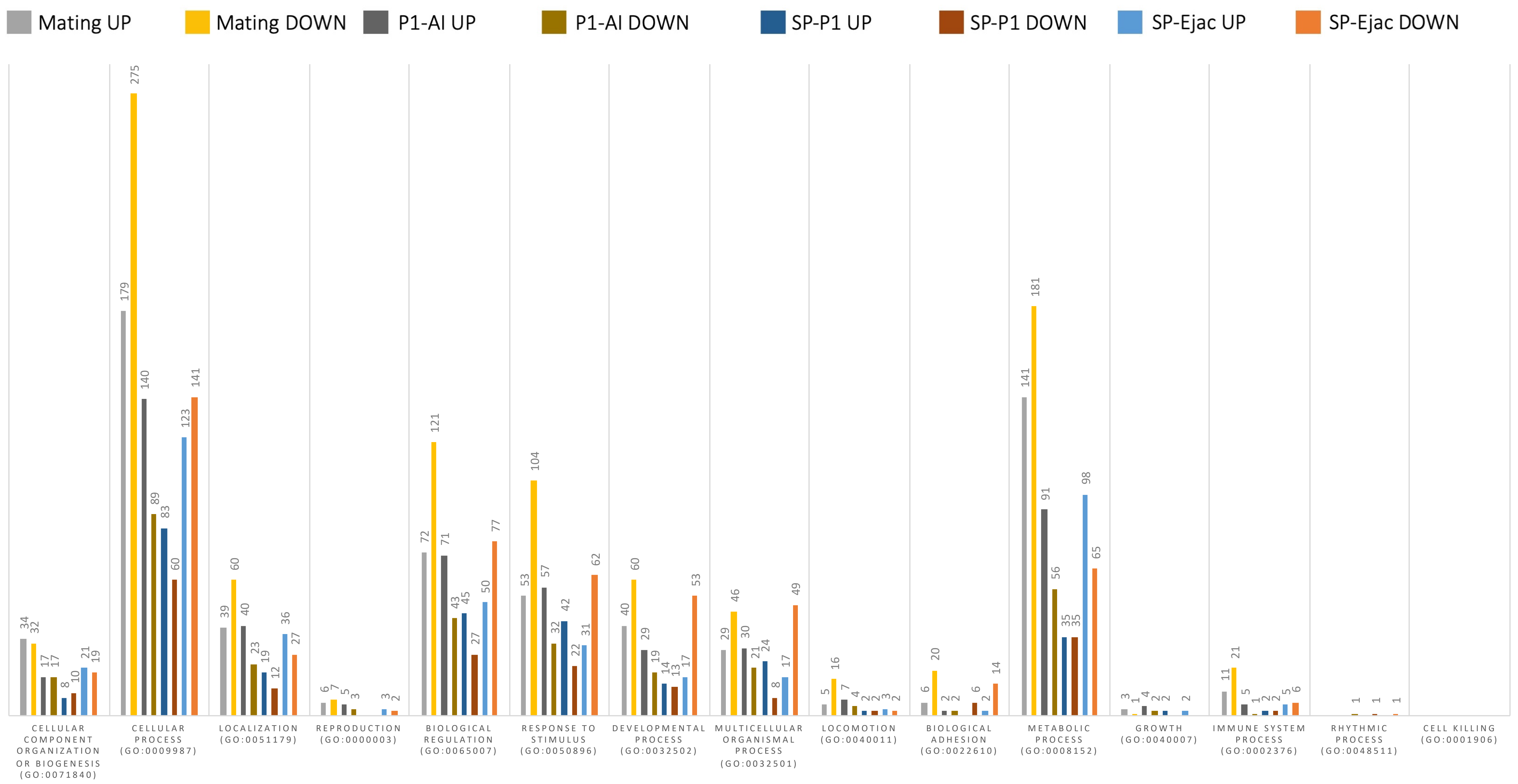

**Supplementary Figure 9.** Biological process in DistEndom

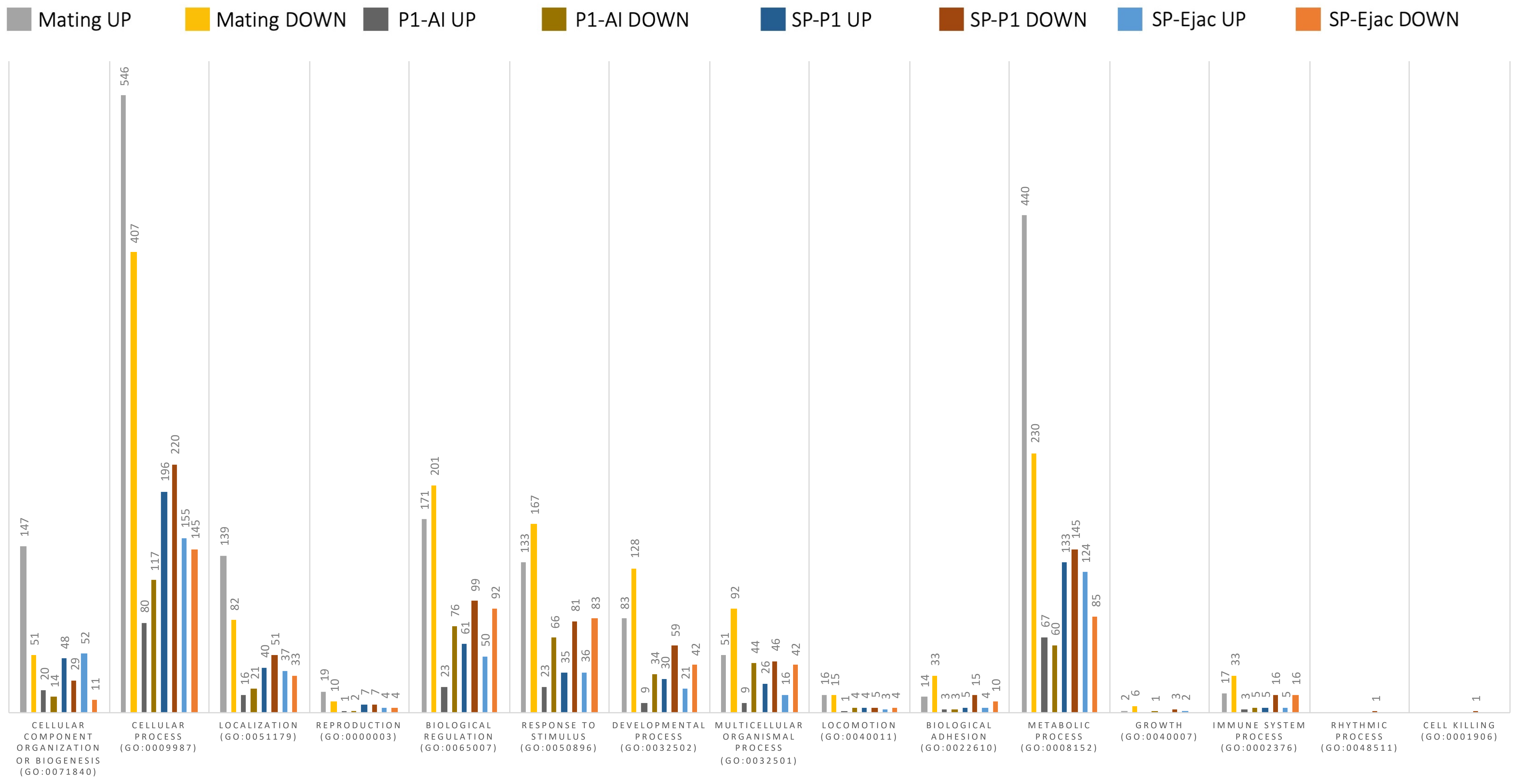

**Supplementary Figure 10.** Biological process in ProxEndom

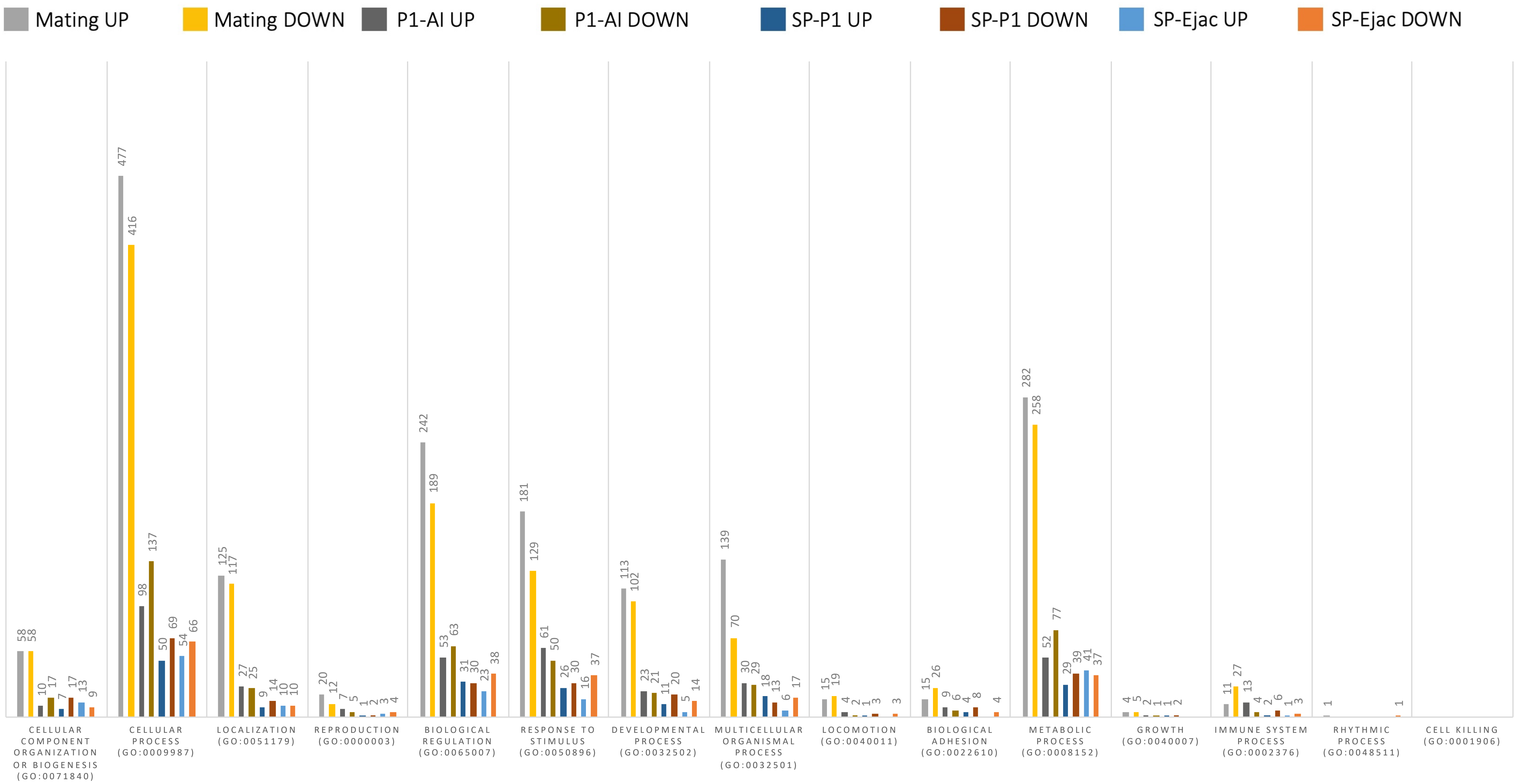

**Supplementary Figure 11.** Biological process in UTJ

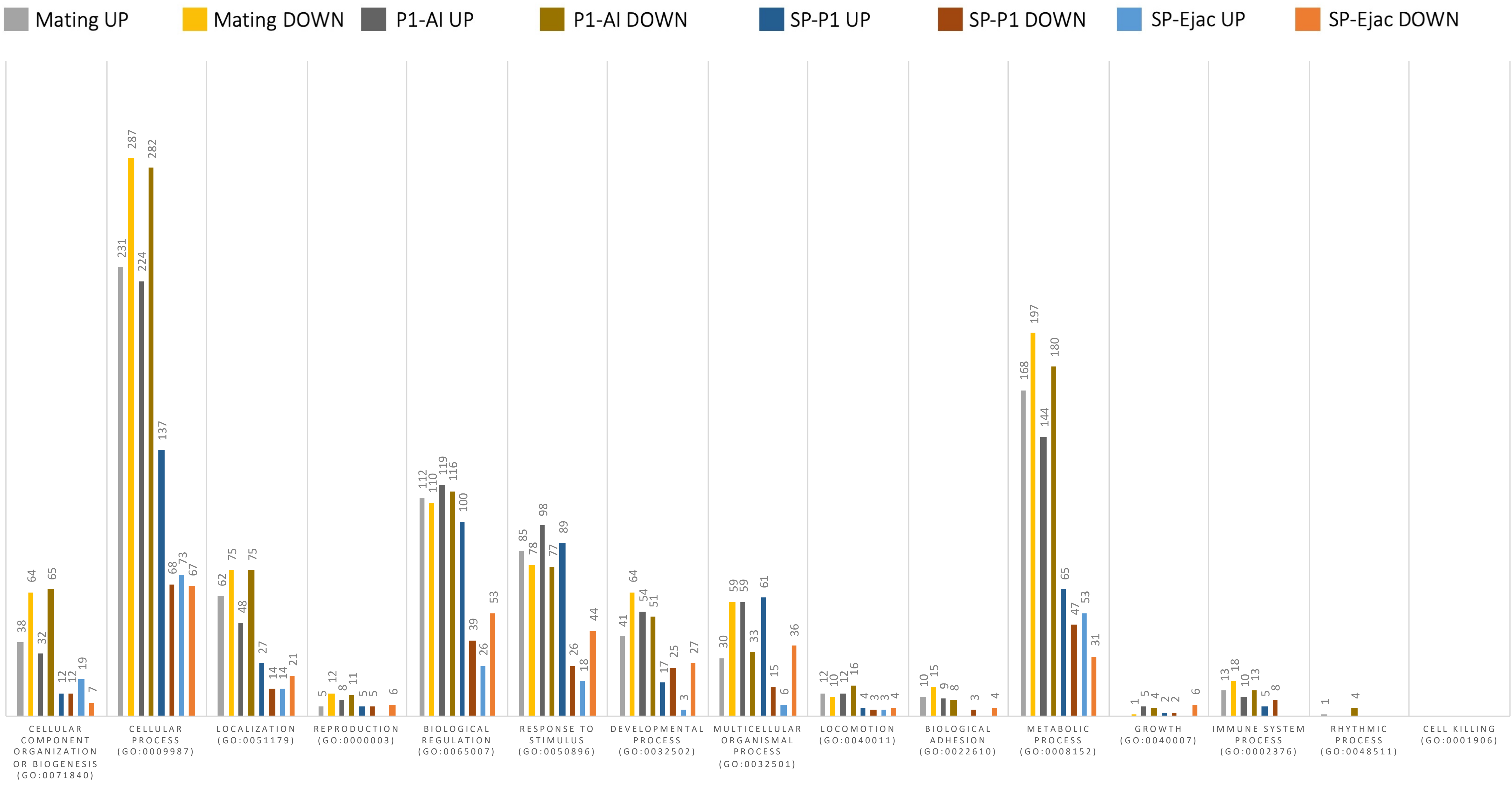

**Supplementary Figure 12.** Biological process in Isth

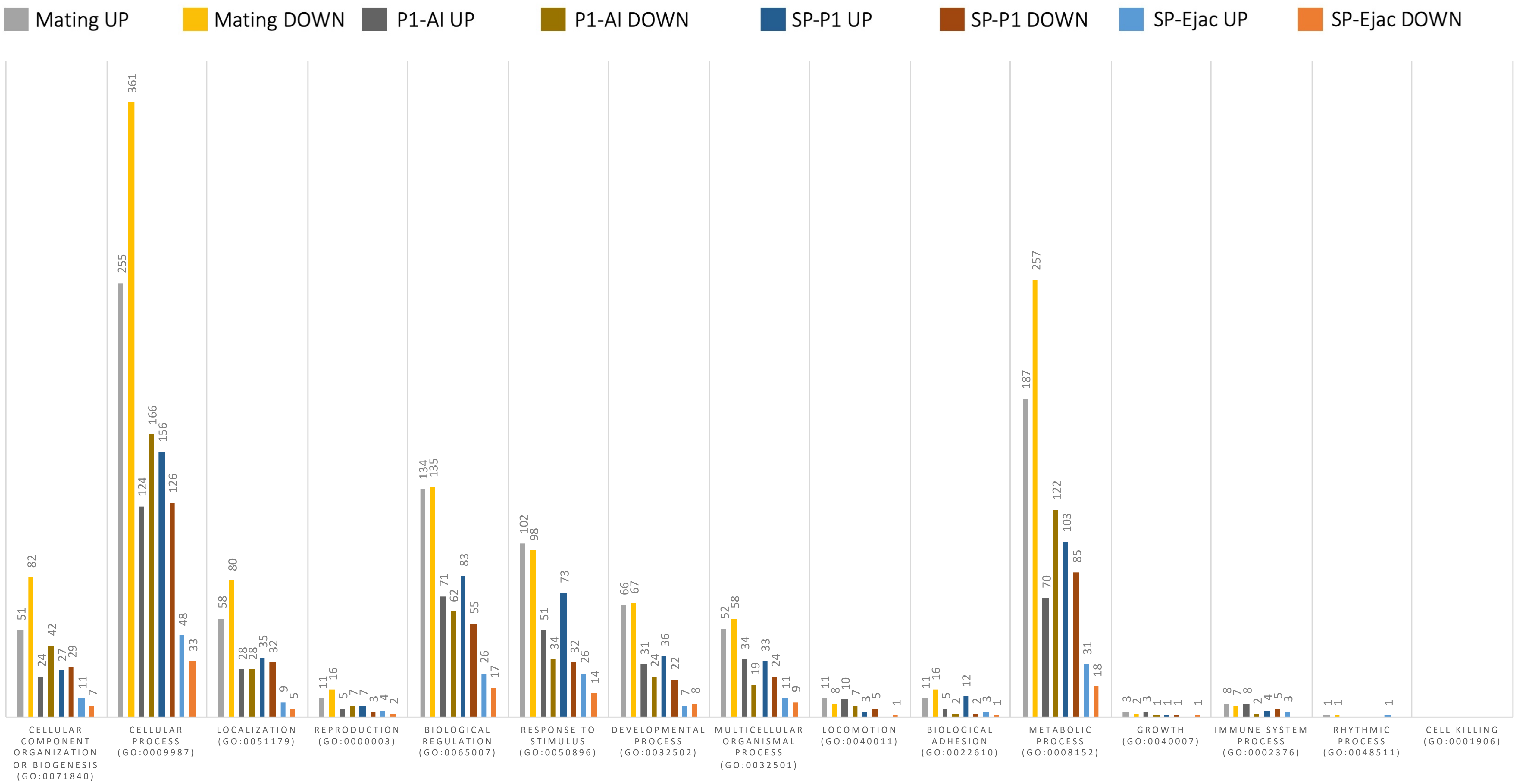

**Supplementary Figure 13.** Biological process in Amp

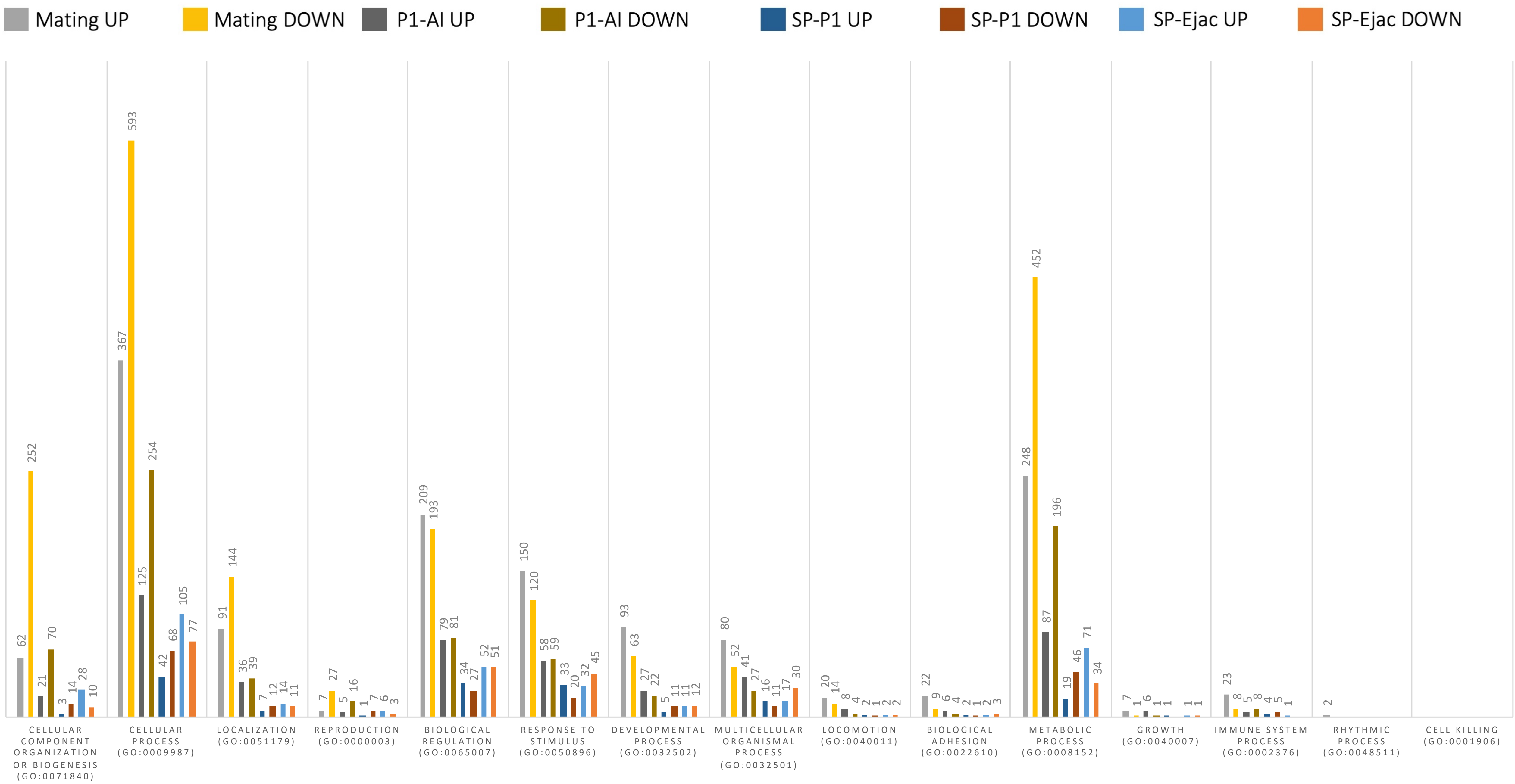

**Supplementary Figure 14.** Biological process in Inf

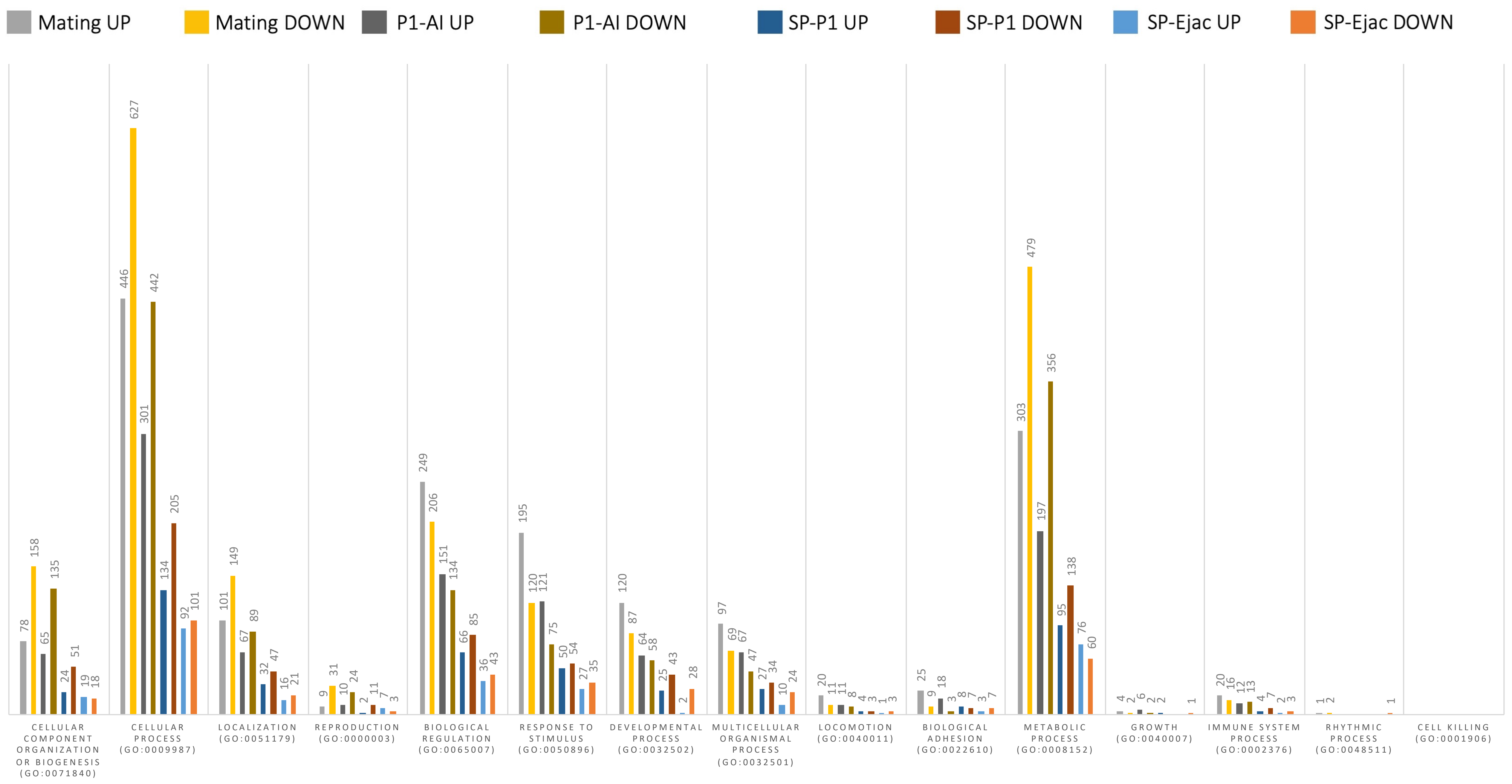

Supplement: Supplementary file 1 — Supplementary information. [file 41598_2020_60810_MOESM1_ESM.pdf]
